# DYRK1A Overexpression Alters Cognition and Neural-Related Proteomic Pathways in the Hippocampus That Are Rescued by Green Tea Extract and/or Environmental Enrichment

**DOI:** 10.3389/fnmol.2019.00272

**Published:** 2019-11-15

**Authors:** Ilario De Toma, Mireia Ortega, Patrick Aloy, Eduard Sabidó, Mara Dierssen

**Affiliations:** ^1^Systems Biology Program, Centre for Genomic Regulation (CRG), The Barcelona Institute of Science and Technology, Barcelona, Spain; ^2^Universitat Pompeu Fabra (UPF), Barcelona, Spain; ^3^Institute for Research in Biomedicine (IRB), The Barcelona Institute of Science and Technology, Barcelona, Spain; ^4^Institució Catalana de Recerca i Estudis Avançats (ICREA), Barcelona, Spain; ^5^Proteomic Unit, Centre for Genomic Regulation, The Barcelona Institute of Science and Technology, Barcelona, Spain; ^6^Centro de Investigación Biomédica en Red de Enfermedades Raras (CIBERER), Valencia, Spain

**Keywords:** proteomics, Down syndrome, hippocampus, EGCG, environmental enrichment, DYRK1A, recognition memory, green tea extract

## Abstract

Down syndrome (DS), caused by trisomy of chromosome 21, is the most common genetic cause of intellectual disability. We recently discovered that green tea extracts containing epigallocatechin-3-gallate (EGCG) improve cognition in mice transgenic for *Dyrk1a* (TgDyrk1A) and in a trisomic DS mouse model (Ts65Dn). Interestingly, paired with cognitive stimulation, green tea has beneficial pro-cognitive effects in DS individuals. Dual Specificity Tyrosine-Phosphorylation-Regulated Kinase 1A (*DYRK1A*) is a major candidate to explain the cognitive phenotypes of DS, and inhibiting its activity is a promising pro-cognitive therapy. DYRK1A kinase activity can be normalized in the hippocampus of transgenic DYRK1A mice administering green tea extracts, but also submitting the animals to environmental enrichment (EE). However, many other mechanisms could also explain the pro-cognitive effects of green tea extracts and EE. To underpin the overall alterations arising upon DYRK1A overexpression and the molecular processes underneath the pro-cognitive effects, we used quantitative proteomics. We investigated the hippocampal (phospho)proteome in basal conditions and after treatment with a green tea extract containing EGCG and/or EE in TgDyrk1A and control mice. We found that *Dyrk1A* overexpression alters protein and phosphoprotein levels of key postsynaptic and plasticity-related pathways and that these alterations were rescued upon the cognitive enhancer treatments.

## Introduction

Down syndrome (DS), caused by trisomy of chromosome 21, is the most common genetic cause of intellectual disability. Recent clinical trials in individuals with DS showed promising pro-cognitive effects of green tea extract containing epigallocatechin-3-gallate (EGCG), a polyphenol of green tea. EGCG also potentiates the effects of cognitive training and increases functional connectivity as shown by fMRI, suggesting an effect of EGCG on neural plasticity (De la Torre et al., 2016). In mice, cognitive training can be reproduced by environmental enrichment (EE), an experimental setup providing mice with a combination of physical activity, learning experiences, and social interaction (Slater and Cao, 2015). EGCG was initially tested in DS because of its properties as inhibitor of the kinase encoded by the Dual Specificity Tyrosine-Phosphorylation-Regulated Kinase 1A (*DYRK1A*), a DS candidate gene located in the 21q22.2 human chromosome region (Duchon and Herault, 2016). The extra copy of the *DYRK1A* increases the expression and the activity of DYRK1A in individuals with DS (Arron et al., 2006). This excessive DYRK1A kinase activity contributes to the DS cognitive disturbances (Altafaj et al., 2001), the motor alterations (Martinez de Lagran et al., 2004), and the characteristic facial features (McElyea et al., 2016), and is also associated with the early onset of Alzheimer’s disease (Sheppard et al., 2012). Supporting these findings, transgenic mice overexpressing Dyrk1A (TG) exhibit defects in neurogenesis, reduced dendritic length and branching, and impaired long-term potentiation (LTP) and long-term depression (LTD), and normalization of its expression levels corrects the cognitive impairments seen in trisomic DS mouse models and in DS individuals (De la Torre et al., 2014). EE also normalizes DYRK1A kinase activity in the hippocampus and rescues neurogenesis alterations and cognitive impairments in TG mice (Pons-Espinal et al., 2013).

However, both EGCG-containing green tea extract and EE induce other pharmacological effects and there is no proof that the improvements of some DS phenotypes are the direct result of DYRK1A inhibition. For instance, both EGCG and EE might act as a radical scavenger and exert indirect effects through activation of transcription factors, signaling regulators, and other enzymes (Liu et al., 2017; Marmol et al., 2017).

To decipher the proteome-wide alterations caused by *Dyrk1A* overexpression and shed light into the mechanism of action of EGCG-containing green tea extracts and EE, we analyzed changes in protein abundances and phosphorylation in mice overexpressing *Dyrk1A* in baseline conditions and under three cognitive enhancer treatments: green tea extract containing EGCG, EE, and their combination. Previous studies have investigated proteomic changes in mouse models of DS (Fernandez et al., 2009; Wang et al., 2009; Ahmed et al., 2012; Ishihara et al., 2014; Nguyen et al., 2018; Vacano et al., 2018) and fetal samples from DS individuals (Bajo et al., 2002; Weitzdoerfer et al., 2002; Shin et al., 2006; Cho et al., 2013; Liu et al., 2016) but this is the first comparing the brain proteomic changes upon pro-cognitive treatments (green tea extract and EE), in *Dyrk1A* transgenic mice.

We show that *Dyrk1A* overexpression leads to broad alterations in protein and phosphoprotein abundance that are not limited to DYRK1A inhibition, and that the cognitive enhancers’ treatments (EE and green tea extracts) restore proteins involved in synaptic and neural plasticity-related pathways. These results will help in developing new combinatorial therapies to boost or prolong current cognitive-enhancement approaches for the treatment of intellectual disabilities.

## Materials and Methods

### Animal Models

All the experiments were performed using 3-month male wild-type (WT) and transgenic mice overexpressing *Dyrk1A* (TG) (Altafaj et al., 2001). Mice were obtained by crossing TG male mice with C57BL6/SJL WT female mice. Mice were reared in standard cages (20 × 12 × 12 cm Plexiglas cage) in groups of two to three animals and maintained under a 12-h light–dark cycle (08:00 h to 22:00 h) in controlled environmental conditions of humidity (60%) and temperature (22 ± 1°C) with *ad libitum* access to food and water. All procedures were approved by the local ethical committee [Comité Ético de Experimentación Animal del PRBB (CEEA-PRBB); MDS 0035P2], and met the guidelines of the local (law 32/2007) and European regulations (EU directive e no. 86/609, EU decree 2001-486) and the Standards for Use of Laboratory Animals No. A5388-01 (NIH). The CRG is authorized to work with genetically modified organisms (A/ES/05/I-13 and A/ES/05/14).

### Experimental Design and Statistical Rationale

At the age of 2 months, TG and WT mice were randomly assigned to the control–non-treated (NT)–and treated groups that were administered with green tea extract (*greentea*) or reared under non-enriched or EE conditions. We used only male mice. We also tested the combination of both green tea extract and EE (*greentea* + *EE*). The behavioral experiments were performed with 144 animals: 38 NT mice (18 TG and 20 WT); 38 treated with green tea extract (18 TG and 21 WT); 36 with EE (16 TG and 18 WT); and 33 treated with green tea extract + EE (16 TG and 17 WT). In each experiment, we included animals of all the experimental groups to avoid batch effects.

For the (phospho-)proteomic analysis, we selected five mice per group tested in the behavioral experiments (WT and TG untreated or treated with green tea extract, EE, and green tea extract + EE; *n* total = 40 mice). The selection of mice was performed avoiding behavioral outliers.

### Pro-cognitive Treatments

The green tea extract (Mega Green Tea Extract, Decaffeinated, Life Extension, United States; EGCG content of 326.25 mg per capsule) was dissolved in drinking water at 0.33 mg/ml corresponding to an average dose of 42 mg/kg per day for 1 month. The solution was freshly prepared every 2–3 days.

One group of mice for each genotype was reared during 1 month in EE conditions. The EE consisted of a spacious Plexiglas cage (55 × 80 × 50 cm) with toys, small houses, tunnels, and platforms of different shapes, sizes, colors, and textures. The arrangement was changed every 3 days to keep novelty conditions. We also stimulated social interactions by housing six to eight mice per cage. To reduce territorial aggressiveness (Haemisch and Gartner, 1997), the animals were reared in the same cage just after the weaning period before they reached sexual maturity.

### Novel Object Recognition

The novel object recognition (NOR) task was performed in both treated and untreated animals at 2 months of age, using a Y-maze consisting of three adjacent arms made of black methacrylate (each arm, 30 × 5 × 6 cm). The task was performed under non-aversive low lighting conditions (50 lux). An overhead camera connected to the video-tracking software (SMART, Panlab) was used to monitor the animals’ behavior. To eliminate odor cues, the arena and the objects were thoroughly cleaned with 10% odorless soap and dried. The location of the objects in the familiarization and test session was counterbalanced between animals. Sniffing time was used as the measure of exploration and was registered by the same experimenter who was blind to genotype and treatment.

The first day, during the habituation session, mice could explore the arena of the Y-maze during 10 min. On the second day, in the familiarization session, the animals explored two identical objects placed at the end of the arms of the Y-maze for 10 min. One hour later, in the test session, mice could explore for 5 min the same arena, but one of the familiar objects was changed for a new one. The exploration time for the familiar (TF) and the new object (TN) during the test phase was recorded. Memory was operationally defined by the discrimination index (DI) calculated as *(TN − TF)/(total exploration time)^∗^100*. We also calculated the distance traveled through the maze, and “spontaneous alternation,” which is the number of alternations in the arm entries on the total number of arm entries.

Significant differences of the DI between experimental groups were assessed using a one-way analysis of variance (ANOVA) taking into consideration the interaction between the genotype and the treatment effect (DI = treatment^∗^genotype). The ANOVA model was implemented using the lm function in the R package stats. Seven contrasts (TG versus WT; and each of the three treatments in both WT and TG mice) were assessed with a *post hoc* correction using the function *glht* from the *multcomp* R package (Hothorn et al., 2008).

We performed a principal component analysis (PCA) with the variables measured in the NOR test (the DI, the percentage of time spent with the novel and familiar object, the distance traveled, and spontaneous alternation), using the R function *prcomp*, scaling the variables to have unit variance and shifting them to be zero-centered.

### Mass-Spectrometry-Based Proteomics

Mice were sacrificed, and the dissected hippocampi were frozen at −80°C. Dyrk1A overexpression was confirmed by western blotting (Supplementary Figure S6). Briefly, 30 ug/sample of protein extract was lysed in canonical 6x Laemmli buffer and loaded on 8% SDS-PAGE gels. Gels were run at a constant 100 volts and transferred to nitrocellulose membranes (iBlot 2, Invitrogen) with default parameters. Filters were blocked with 10% skim milk powder in Tris-bufferedsaline, pH 7.0, 0.1% Tween 20 (TBS-T). Blocked filters were incubated overnight with anti-Dyrk1A (RR.7) Antibody 1:1000 (Santa Cruz) and anti-actin 1:5000 (A2066, Sigma Aldrich), and 1 h with IRDye 800CW secondary antibodies for fluorescent detection with the 800 nm channel of Odyssey^®^ Imaging Systems. Densitometric differences were assessed statistically with a Wilcoxon rank sum test. For the analysis of the hippocampal proteome, we selected five mice per experimental condition. The sample was randomly selected from inside the 1.5^∗^(interquartile range) in the NOR task in order to remove outliers that would have skewed our data. This led to a reduction of cohort variability (from 1.4 to 7.3 depending on the group) but allowed us to focus on prototype mice, avoiding animals with extreme behaviors. The hippocampi of the selected mice were processed at the same time, homogenized with a RIPA-modified buffer (50 mM tris–HCl, pH 7.5, 150 mM NaCl, 1 mM EDTA, 1% NP-40, and 0.1% sodium deoxycholate with the addition of 5 mM b-glycerophosphate, 10 mM sodium fluoride, 10 mM sodium orthovanadate, and protease inhibitors from the Complete Protease Inhibitor Cocktail Roche).

Samples were sonicated using Bioruptor^®^ (Diagenode) for 5 min with 30 on/off cycles, maintaining the samples on ice, and were centrifuged for 10 min at 10,000 rpm at 4°C. Proteins from the supernatants were precipitated overnight at -20°C by adding a volume of ice-cold acetone in sixfold excess. The acetone-precipitated proteins were solubilized in a denaturation buffer (6 M urea and 200 mM ammonium bicarbonate in water). Final protein content was quantified using the BCA assay (Pierce). Proteins were reduced with dithiothreitol (DTT, 10 mM, 37°C, 60 min), and alkylated with iodoacetamide (IAM, 20 mM, 25°C, 30 min). Then, samples were diluted with 200 mM ammonium bicarbonate up to 2 M urea, digested overnight with Lys-C at 37°C, and then diluted twofold again and digested overnight with trypsin at 37°C. Peptides were desalted using a C18 MicroSpin 300A silica column (The Nest Group, Inc.), evaporated to dryness using a speedvac, and dissolved in 30 μl of 0.1% formic acid in water.

### Titanium Dioxide (TiO_2_) Phosphopeptide Enrichment

Phosphopeptides were enriched using titansphere chromatography. Briefly, tryptic peptides were desalted and completely evaporated to dryness, and then they were dissolved with 100 μl of Loading Buffer [80% ACN (vol/vol) and 6% TFA (vol/vol)] at ∼1 μg/μl concentration of peptides. Samples were passed through a constricted TiO_2_-loaded spin tip, previously equilibrated with Loading Buffer, applying 2 × 50 μl and using a centrifuge at ∼50 *g* in order to achieve a complete binding. TiO_2_ spin tip was washed once with 50 μl of Loading Buffer and once with 50 μl of Washing Buffer [50% ACN (vol/vol) and 0.1% TFA (vol/vol)]. Finally, phosphopeptides were eluted from the TiO_2_ spin tip with 30 μl of Elution Buffer (85% NH_3_-H_2_O, pH 11.0) into a tube that contains 30 μl of 20% formic acid. Second elution was made in the same tube with 3 μl of Elution Buffer 2 [(80% ACN (vol/vol) and 2% formic acid (vol/vol)]. The eluted phosphopeptides were evaporated to dryness and dissolved with 0.1% formic acid in water for being analyzed by mass spectrometry (MS).

### Liquid Chromatography-Tandem Mass Spectrometry

For each sample, 1 μg of tryptic peptides from digested hippocampal tissue and phospho-enriched peptides from 100 μg of the same tissue were injected in an LTQ-Orbitrap Velos Pro mass spectrometer (Thermo Fisher Scientific) coupled to a nano-LC (EASY-nLC, Proxeon). Nano-LC was equipped with a reversed-phase chromatography column of 25 cm with an inner diameter of 75 μm, packed with 3 μm C18 particles (Nikkyo Technos, NTCC-360/75-3-25L), and a Nano Trap Column Acclaim PepMap100 100 μm × 2 cm C18, 5 μm, 100 A (Thermo, 164199). Chromatographic gradients started at 93% of buffer A and 7% of buffer B with a flow rate of 250 nl/min during 5 min and linearly changed to 65% buffer A and 35% buffer B after 240 min. After each analysis, the column was washed for 16 min with 90% buffer A and 10% buffer B (buffer A: 0.1% formic acid in water; buffer B: 0.1% formic acid in acetonitrile).

The mass spectrometer was operated in positive ionization mode with the nanospray voltage set at 2.2 kV and the source temperature at 250°C. Ultramark 1621 for the FT mass analyzer was used for external calibration prior to the analyses. The background polysiloxane ion signal at *m/z* 445.1200 was used as lock mass. The instrument was operated in data-dependent acquisition (DDA) mode with 1 microscan at a resolution of 60,000 at 400 *m/z* and survey scans were recorded over a mass range of *m/z* 350-2000 with detection in the Orbitrap mass analyzer. Auto gain control (AGC) was set to 106, dynamic exclusion was set at 60 s, and the charge-state filter disqualifying singly charged peptides for fragmentation was activated. Following each survey scan, the top 20 most intense ions with multiple charged ions above a threshold ion count of 5000 were selected for fragmentation at a normalized collision energy of 35%. Fragment ion spectra produced via collision-induced dissociation (CID) and CID multistage activation (CID MSA) for proteome and phosphoproteome, respectively, were acquired in the linear ion trap, and AGC was set to 5 × 10^4^, and an isolation window of 2.0 *m/z*, an activation time of 0.1 ms, and a maximum injection time of 100 ms were used.

### MS Data Analysis

Acquired mass spectra were processed using the *MaxQuant* computational platform version 1.5.2.8 (Cox and Mann, 2008; RRID:SCR_014485). The MS2 spectra were searched by using the Andromeda search engine (Cox et al., 2011) against the Uniprot sequence database for *Mus musculus* (17,263 forward entries; version from July 2015). The search included cysteine carbamidomethylation as a fixed modification, and N-terminal protein acetylation and methionine oxidation as variable modifications. In the case of phosphoproteome analysis, phosphorylation on serine, threonine, and tyrosine was also added as variable modifications. We allowed a maximum of two mis-cleavages, 4.5 ppm as mass tolerance for precursor ions, and 0.5 Da as mass tolerance for fragment ions. FDR was set to 1% at the peptide and protein level, and protein identification required at least one unique or razor peptide per protein group.

The *MaxQuant* algorithm was used to retrieve accurate extracted ion currents (XICs) per peptide feature for quantification purposes. Areas under the curve for each peptide were calculated and later used to estimate protein intensities during the statistical analysis. Statistical analysis was performed using the R package *MSstats* (Choi et al., 2014) version 2.6.0 (RRID:SCR_014353). In some of the experimental groups, one of the five biological replicates was excluded from the analysis, when the number of peptides identified was substantially lower (e.g., 1000 peptides) than the average of peptides identified in the whole experiment. One replicate was excluded from the *TG.greentea* + *EE* and *WT.EE* groups in proteome analysis, and another replicate was excluded from the *TG.greentea* and *WT.greentea* + *E*E groups in the phosphoproteome analysis. To ensure high confidence in our quantitative data, only peptides observed at least in three of the five biological replicates (or at least in two when we remained only with four biological replicates) were used, and no imputation of missing values was performed.

### Differentially Expressed Proteins and Phosphopeptides

Differential expression analysis was performed with the *MSstats* package that is based on a family of linear mixed-effect models (Choi et al., 2014). Downstream bioinformatics analysis (e.g., enrichment analyses) was performed on proteins and phosphopeptides that showed a significant change in abundance with a *Benjamini* adjusted *p*-value lower than 0.05 and a log_2_(fold change) (log_2_FC) greater than 0.3 or lower than -0.3. For the phosphoproteomic analysis, only phosphorylation sites with a localization probability of 0.5 or higher were considered to avoid uncertain sites. The proteins or phosphopeptides uniquely present in one condition of the ones compared were added to the lists of differentially abundant proteins and phosphopeptides. A peptide was defined as “absent/low abundant” when it was detected in less than three out of five biological replicates for a given condition (or less than two out of five biological replicates). Since blood contamination is a common problem in sample collection from dissected tissues, proteins belonging to the GO-term cell component “blood microparticle” were filtered out from all datasets before proceeding with downstream analyses.

We calculated the following contrasts for each of the three treatments (TG: TG mice; WT: wild-type mice; NT: non-treated mice; T: treated mice):

• *TG.NT-WT.NT* (deregulated proteins in untreated TG mice);
• *TG.T-TG.NT* (proteins responding to the treatments in TG mice);
• *WT.T-WT.NT* (proteins responding to the treatments in WT mice);
• *(TG.T-TG.NT)–(WT.T-WT.NT)* (interaction: proteins responding differently to the treatments in TG compared to WT mice).

For each protein/phosphopeptide whose abundance was significantly changing in the *TG.NT-WT.NT* contrasts, we first calculated the genotype gap as the log_2_FC obtained in the contrast *TG.NT-WT.NT*. Thereafter, we computed the “treatment gap” as the fold change observed in the *TG.T-WT.NT* contrast. We therefore calculated the “percentage of recovery” based on the fraction of recovered abundance—in protein or phosphopeptides levels—after treatment—given by the difference between the genotype gap and the treatment gap—on the genotype gap: (genotype gap) - (treatment gap)/(genotype gap).

Note that once *WT.NT* is set to 0 [log2(1) = 0]:

• *TG.NT-WT.NT* contrast can be simplified by TG.NT – log2(1) = TG.NT-0 = TG.NT
• *TG.T*-*WT.NT* = Levels impaired at the basal state + levels after treatment = *(TG.NT-WT.NT)* + *(TG.T-TG.NT)* = *−0* + *TG.T* = *TG.T*

And so:

*(TG.NT*-*TG.T) TG.NT* = = *[(TG.NT-WT.NT)* − *(TG.NT-WT.NT)* + *(TG.T* + *TG.NT)]/(TG.NT-WT.NT).*

The fraction of recovery could go from 0 (no recovery) to 1 (100% recovery). A value > 1 indicates overcorrection. A value < 0 indicates an impairment.

We considered as “rescued protein” those proteins significantly changing in the TG.NT-WT.NT contrasts, with a% of recovery from 50 to 150%; “overcorrected proteins,” the ones with a% of recovery higher than 150; “not sufficiently rescued,” those proteins with a% of recovery between −50% and 50%; and “impaired proteins,” those with a% of recovery < −50%.

With similar calculations, we computed the “percentage of impairment” by using instead of the *TG.NT-WT.NT* contrasts, its reverse *WT.NT-TG.NT*, in order to calculate how much the treatment in the WT was reducing the differences between TG and WT.

### DYRK1A Interactors

DYRK1A interactors were taken from the mammalian verified interactors reported in previous literature (Aranda et al., 2011; Duchon and Herault, 2016; Guard et al., 2019; Roewenstrunk et al., 2019). The significance of the overlaps between DYRK1A interactors and the differentially abundant proteins, or between DYRK1A interactors and the proteins with differentially abundant phosphopeptides, was assessed using a Fisher exact test.

### Network Analysis

Lists of differentially abundant and phosphopeptides were expanded to build a protein–protein interaction (PPI) network expanded to their direct interactors.

Specifically, for building the network, we used the expanded list of proteins deregulated in the *TG.NT-WT.NT* contrast. For the expansion, we used a list of bona fide physical interactors mainly coming from the STRING (version 10) (Szklarczyk et al., 2015) and an internal database from *Interactome3d* version 2017_01 (Mosca et al., 2013). We only considered interactions with a very high score (> 0.9) in *STRINGdb* and *IMEx* index (Orchard, 2012; Orchard et al., 2012).

Graph visualization was performed with the igraph R package (Csardi and Nepusz, 2005) using the *Davidson Harel layout algorithm* (Hadany and Harel, 2001). In order to calculate the odds of the observed number of interactions compared to the expected ones, we used the *ppi_enrichment* function from the *STRINGdb* package (Pradines et al., 2005).

To compute differences in the properties of the network between different sub-graphs of the network (i.e., differences in node degree), we used a permutation test with 1000 permutations, recalculating at each step the given property on a random subset of the same size.

### Enrichment Analyses

Protein identifiers were annotated using the R packages *UniProt.ws* and *biomaRt* (Durinck et al., 2009) (RRID:SCR_002987). We assessed the significance of the overlaps using a Fisher’s exact test; *p*-values for the right tail are obtained directly using hypergeometric distribution considering as background the detected proteins for each contrast. For Gene Ontology Enrichment analysis, we used the *clusterProfiler* R package (Yu et al., 2012) (RRID:SCR_016884).

### Transcription Factor Prediction Analysis

Transcription factor enrichment analyses were performed using the *iRegulon* plugin (version 1.3) implemented in *Cytoscape* (Janky et al., 2014). The *iRegulon* plugin allows the identification of transcription factors using motif discovery in a set of proteins. Proteins changing in abundance were analyzed in two different sets of networks depending on the sign of the fold change in logarithmic scale. Thus, one network corresponding to proteins with a log_2_FC > 0 (increased abundance) and another one with proteins with a log_2_FC < 0 (decreased abundance) were created. Default values of the plugin were maintained except for the database that was changed to *M. musculus*. The enrichment of these genes was determined in each of the N motif-based rankings using the area under the cumulative recovery curve (AUC), whereby the AUC is computed in the top of the ranking (default set to 3). The AUC values are normalized into a normalized enrichment score (NES) on which we set a default cutoff of 3.0, corresponding to a false discovery rate (FDR) between 3% and 9% (Janky et al., 2014).

### Identification of Significant Hubs

We used the *poweRlaw* R package (Gillespie, 2015) to analyze the heavy tail distribution of interactions. We compared the power-law, Poisson, exponential, and lognormal distribution. Thereafter, we used the fitted distribution for calculating the *p*-values associated with each protein corresponding to encountering by chance a higher number of interactions per protein than the given protein, setting the threshold to define hub at *p* < 0.05.

### Correlation With Behavioral Data

The obtained PC1 values (learning-related variables) per animal used in the MS analysis were correlated with protein and phosphopeptide levels. Based on the distribution of *rho* values (Spearman correlation), a cutoff of ± 0.4 (i.e., *rho* > 0.4 or *rho* < -0.4) was applied obtaining two lists of proteins: (i) a list of proteins whose abundances were highly correlating (or anti-correlating) with PC1 and (ii) a list of proteins with phosphopeptides levels that were correlating (or anti-correlating) with PC1.

### Data Availability Statement

The MS proteomics dataset has been deposited to the *ProteomeXchange* Consortium via the *PRIDE* (Vizcaino et al., 2016) partner repository (RRID:SCR_003411) with the dataset identifier *PXD010689*. A repository with the R markdown file with the code to fully reproduce the analysis is present at the repository: https://bitbucket.org/ilario_de_toma/proteomicsdyrk1a.egcg.ee/src.

## Results

### *Dyrk1A* Overexpression Affects Differentially the Hippocampal Proteome and Phosphoproteome

We quantified protein abundances and phosphorylation levels using quantitative MS-based proteomics (LC-MS/MS) on whole proteome extracts from the hippocampi of TgDyrk1A (TG) and WT mice. In total, we identified 3001 proteins and 3437 phosphopeptides belonging to 1134 phosphorylated proteins. We fitted the data into a linear model to evaluate the changes related to the genotype, to the treatments, and their interaction. Setting a threshold of adjusted *p*-value and fold change of *p* < 0.05, and | log_2_FC| > 0.3, we found that *Dyrk1A* overexpression in TG hippocampus resulted in significant changes in the abundance of 98 proteins (51 upregulated and 47 downregulated) compared to WT. Dysregulated proteins were enriched in targets of 10 transcription factors (Table 1), suggesting that the effect DYRK1A on protein abundance could be due to the activation of transcription factors.

**TABLE 1:** Summary statistics for the proteomic study.

	**Detected proteins (*n*)**	**Upregulated (*n*)**	**Present in TG, low/absent in WT**	**Downregulated (*n*)**	**Low/absent in TG, present in WT**	**DYRK1A interactors**	**DYRK1A interactors (in direct interactors)**
TG.NT-WT.NT	2685	33	18	24	23	Hras	Braf, Dnm1, Psen1, Efnb1, Efnb2, Grb2, Rbl2, Sirt1
WT.greentea-WT.NT	2695	43	18	37	17	Capn1, Eif4e, Hras, Tsc1, Tollip	Braf, Grin2a, Dnm1, Stat3, Psen1, Efnb1, Efnb2, Grb2, Eif4ebp1, Amph
WT.EE-WT.NT	2758	28	34	19	43	Hras, Rbm39, Nup155, Tollip, Myo1c	Braf, Stat3, Efnb1, Efnb2, Ywhaq, Sh3gl2, Sirt1
WT.greentea + EE-WT.NT	2690	38	22	34	16	Cnbp, Hras, Huwe1, Pgam5, Ablim1	Braf, Stat3, Brca1, Eif4e, Rest, Sirt1, Rai14
TG.greentea-TG.NT	2650	23	12	40	23	Lrch1, Fntb, Spred1	Dnm1, Stat3, Psen1, Efnb1, Efnb2, Epha2, Btrc, Grb2, Rbm39
TG.EE-TG.NT	2680	35	27	32	19	Ech1, Spred1, Tsc1	App, Dnm1, Psen1, Efnb1, Efnb2, Bicd2, Hipk2
TG.greentea + EE-TG.NT	2756	37	46	65	56	Cdkn1b, G6pdx, Srsf7, Tsc1	Ccnd1, Psen1, Nfatc2, E4f1, Hipk2
Rescued by the green tea extract	33	17	3	7	6		Dnm1, Psen1, Efnb1, Efnb2, Grb2
Rescued by EE	34	13	4	8	9	Hras	Braf, Dnm1, Psen1, Efnb1, Efnb2, Rbl2
Rescued by the combined treatment	46	15	7	12	12		Psen1

*Proteins were considered differentially expressed if having an adjusted *p*-value lower than 0.05 and a log_2_ (fold change) greater than 0.3 or lower than -0.3. Further details are provided in Supplementary Tables S1–S10.*

**FIGURE 1:**
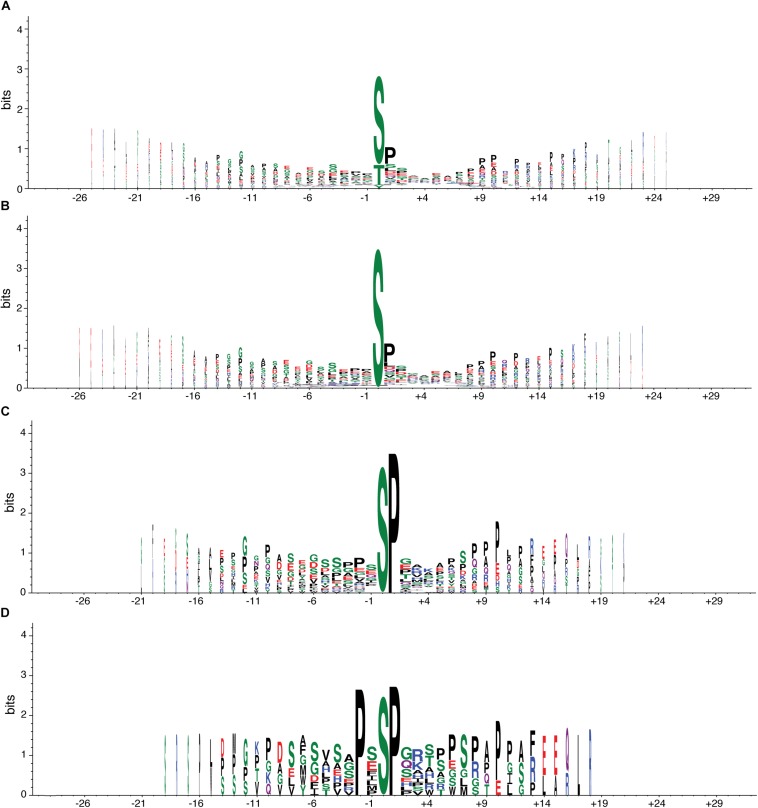
Consensus motif of phosphopeptides altered in transgenic mice overexpressing *Dyrk1A*. Graphical representation of the sequence conservation of amino acids in phosphopeptides upregulated (or exclusively present) in TG mice. The overall height of the stack indicates the sequence conservation at a given position, while the height of symbols within the stack indicates the relative frequency of each amino acid at that position. Polar amino acids are in green, neutral ones in purple, basic ones in blue, acidic ones in red, and hydrophobic ones in black. Stack width is scaled by the fraction of amino acids in the column. **(A)** Sequence logo with all phosphopeptides (98) upregulated (or exclusively present) in TG mice compared to WT mice. **(B)** All phosphopeptides in **A** with a phosphorylated serine, the most common site of phosphorylation (82). **(C)** All phosphopeptides in **B** with a proline in position + *1*, the most common + *1* site (35). **(D)** All phosphopeptides in **B** with a proline in position -*2*, the most common -*2* site (11).

At the phosphoproteome level, we identified 98 phosphopeptides—corresponding to 90 proteins—that increased their abundance in TG mice compared to their WT counterparts, and 105 phosphopeptides—mapping to 100 different proteins—that decreased their abundance (Tables 2, 3). To understand whether DYRK1A kinase activity could explain part of the changes in the phosphopeptide levels, we analyzed the phosphorylation motifs of the deregulated phosphopeptides creating a sequence logo (Figure 1A). We found that 82 (out of the 98 phosphopeptides that increased their abundance in TG hippocampus, see above) had a phosphorylated serine (Figure 1B), with 35 of them having also a proline in position + 1 (Figure 1C), and 11 having a proline in position -2 (Figure 1D). PXSP is a known consensus motif for the ERK1/2/MAPK pathway, which has previously been linked with learning and memory (Peng et al., 2010), and DYRK1A, being a proline directed kinase, has an optimal phosphorylation sequence similar to ERK2. We also detected two phosphopeptides belonging to the SGIP1 and SHANK3 proteins, phosphorylated in the consensus motif RPX(S/T)P, the canonical phosphorylation motif described for DYRK1A (Himpel et al., 2000).

**TABLE 2:** Summary statistics for the phospho-proteomic study.

	**Detected proteins (*n*)**	**Upregulated (*n*)**	**Present in TG, low/absent in WT**	**Downregulated (*n*)**	**Low/absent in TG, present in WT**	**DYRK1A interactors**	**DYRK1A interactors (in direct interactors)**
TG.NT-WT.NT	1409 (722)	16 (16)	82 (74)	23 (22)	82 (78)	Map1b, Nf1, Aarsd1, Srsf2, Dpysl3, Eif4b, Srsf7, Smad3, Ablim1, Nos1ap, Gys1, Ap3b1, Synj1	Trp53, Polr2a, Mapt, Ap2a1, Efnb1, Efnb2, Ywhaq, E2f5, E2f4, Tardbp, Sirt1, Dynll2
WT.greentea-WT.NT	1498 (761)	27 (27)	130 (112)	20 (19)	51 (45)	Etl4, Mapt, Map1b, Grin2a, Amph, Eif4b, Ablim1, Luzp1, Prkar1a, Gsk3b, Gys1	Capn2, Fhl2, Prkaca, Ap2a1, Dnm1, Stat3, Eps15, Eif4a1, Eif4e, Kpnb1, Btrc, Sirt1
WT.EE-WT.NT	1574 (789)	43 (37)	193 (157)	34 (32)	57 (50)	Etl4, Dpysl2, Mapt, Map1b, Sept4, Prkacb, Dpysl3, Eif4b, Srsf7, Ablim1, Luzp1, Nos1ap, Phf6, Gys1, Synj1	Ctbp1, Trp53, Dnm1, Eps15, Eif4a1, Ywhag, Eif4e, Smarca4, Tardbp, Sirt1, Dynll2, Prkar1a, Park2
WT.greentea + EE-WT.NT	1674 (827)	24 (24)	211 (175)	27 (27)	122 (99)	Etl4, Dpysl2, Mapt, Map1b, Prkacb, Nf1, Ppm1g, Srsf2, Dpysl3, Amph, Eif4b, Ablim1, Nos1ap, Phf6, Ppp1r2, Gys1, Synj1	Capn2, Trp53, Polr2a, Map2k1, Dnm1, Stat3, Psen1, Tpm1, Eif4a1, Eif4e, Mapk1, Kpnb1, Creb1, Grb2, Wasl, Tardbp, Sirt1, Dynll2
TG.greentea-TG.NT	1691 (832)	21 (20)	225 (194)	27 (26)	102 (89)	Dpysl2, Mapt, Map1b, Sept4, Grin2a, Tnks1bp1, Aarsd1, Srsf2, Dpysl3, Eif4b, Srsf7, Smad3, Eif2b5, Anln, Ablim1, Luzp1, Phf6, Prkar1a, Ppp1r2, Gys1, Ap3b1, Synj1	Fhl2, Trp53, Prkaca, Polr2a, Ap2a1, Map2k1, Dnm1, Traf2, Eps15, Psen1, Efnb1, Efnb2, Eif4a1, Ywhag, Eif4e, Mapk1, Dynll1, Ywhaq, Kpnb1, Creb1, Btrc, E2f5, Camsap2, E2f4, Sirt1, Gsk3b, Park2
TG.EE-TG.NT	1427 (743)	15 (15)	82 (73)	29 (25)	65 (60)	Dpysl2, Mapt, Map1b, Sept4, Prkacb, Enah, Aarsd1, Huwe1, Smad3, Anln, Nos1ap, Ppp1r2, Synj1	Stxbp1, Fhl2, Ap2a1, Dnm1, Stat3, Eps15, Psen1, Dynll1, Btrc, E2f5, Srsf2, E2f4, Gsk3b
TG.greentea + EE-TG.NT	1356 (702)	13 (13)	55 (51)	17 (17)	100 (92)	Mapt, Map1b, Sept4, Tnks1bp1, Nf1, Aarsd1, Srsf2, Huwe1, Srsf7, Smad3, Ablim1, Nos1ap, Phf6, Gsk3b, Synj1	Fhl2, Prkaca, Polr2a, Ap2a1, Map2k1, Dnm1, Traf2, Stat3, Psen1, Efnb1, Efnb2, Eif4a1, Eif4e, Dynll1, Ywhaq, Kpnb1, Btrc, E2f5, E2f4, Dynll2, Tollip
Rescued by green tea extract	87 (81)	10 (12)	26 (28)	4 (7)	47 (50)	Map1b, Aarsd1, Eif4b, Srsf7, Smad3, Gys1, Synj1	Efnb1, Efnb2, Ywhaq, E2f5, Srsf2, E2f4, Dynll2
Rescued by EE	67 (64)	11 (13)	24 (28)	7 (10)	25 (29)	Map1b, Aarsd1, Srsf2, Smad3, Nos1ap, Ap3b1, Synj1	Polr2a, Ap2a1, E2f5, E2f4, Dynll2
Rescued by the combined treatment	65 (60)	7 (11)	31 (32)	7 (7)	20 (24)	Map1b, Nf1, Aarsd1, Srsf2, Srsf7, Smad3, Nos1ap, Synj1	Polr2a, Ap2a1, E2f5, E2f4, Dynll2

*Numbers refer to phosphopeptides. The corresponding number of proteins is reported in brackets. Phosphopeptides were considered differentially expressed if having *p*-value lower than 0.05 and a log_2_ (fold change) greater than 0.3 or lower than −0.3. Proteins containing at least a differentially expressed phosphopeptide were called “differentially phosphorylated.” Further details are provided in Supplementary Tables S11–S20.*

**TABLE 3:** Transcription factors predicted by *iRegulon*.

**Upregulated proteins (*TG.NT-WT.NT*)**			**Downregulated proteins (*TG.NT-WT.NT*)**		
	
**Transcription factor**	**Targets**	**NES**	**Transcription factor**	**Targets**	**NES**
Srf	10	5.1	Msx1	17	5.1
Cebpa	8	4.7	Nkx2-2	13	4.9
Arntl	9	4.5	Ddx43	10	4.7
Nr2c2	10	4	Tcf3	10	4.6
			Smc3	20	4.6
			Foxo1	12	4.1

*The *Targets* column indicates the number of proteins predicted as targets of the transcription factors. The *NES* (normalized enrichment score) column indicates the score of the enrichment prediction. Only transcription factors with a NES higher than four were considered.*

Interestingly, the proteins changing in abundance in transgenic mice were different from the protein changing their phosphorylation, with only 5% of the dysregulated proteins changing both their levels and phosphorylation.

### The (Phospho-)Proteomic DYRK1A Network in Hippocampus Reveals Alterations in Synaptic and Neurogenesis Proteins

**FIGURE 2:**
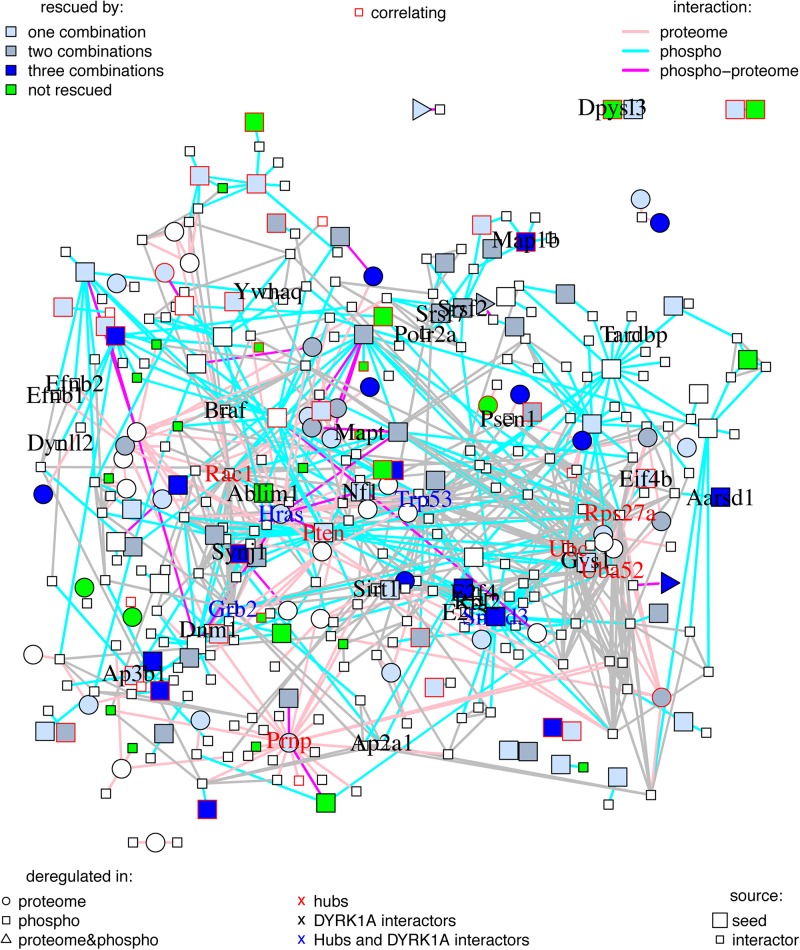
Protein–protein interaction network of proteins changing in TG mice overexpressing *Dyrk1A* compared to WT mice. Graph where the nodes correspond to proteins changing in TG mice overexpressing *Dyrk1A* (*seeds*) and their direct interactors, and edges correspond to known protein–protein interactions. *Seed* proteins are represented as bigger nodes, circles correspond to protein changing their abundances, squares to proteins changing their phosphorylation, and triangles to proteins changing both their abundance and their phosphorylation levels. Proteins with abundance restored by the treatments are colored in blue (light blue are rescued just by one of the treatment combinations, medium blue by two combinations, and darker blue by the three treatments). Proteins changing in TG mice but not rescued, impaired, or overcorrected are in green. Interactions involving a protein changing its abundance are represented in pink, those involving a protein changing its phosphorylation level are in cyan, and those involving protein changing both abundance and phosphorylation are in purple. The thickness of the edge is proportional to the interaction score. Nodes corresponding to proteins whose abundances or phosphorylation correlates with cognition are depicted with a red frame. Names of DYRK1A interactors and hubs are printed out. Black: DYRK1A interactors. Red: hubs. Blue: both DYRK1A interactors and hubs.

To gain more insight into the proteome and phosphoproteome alterations induced by *Dyrk1A* overexpression, we constructed a PPI network with the 262 proteins dysregulated—both at the level of abundances and phosphorylation—in TG hippocampus (*seed* proteins, represented in bigger size in Figure 2), with the primary interactors (from the STRING database) ending up with 372 interacting proteins (Figure 2). The proteins of this network were interacting more than expected by chance, suggesting that the significant changes in abundance and phosphorylation in TG hippocampal proteins are functionally related. When only considering the *seed* proteins, the proteins changing their phosphorylation state were the most connected nodes of the network: we found 20 times an interaction between two proteins changing their phosphorylation (represented as cyan edges connecting two squares in Figure 2); nine times an interaction between proteins only changing their abundance (pink edges connecting two circles); and 21 times an interaction between proteins changing abundance and proteins changing phosphorylation levels (represented by magenta edges connecting a circle with a square, a circle with a triangle, or a square with a triangle, respectively).

We detected 50 interactions, versus 14 expected for *seed* proteins, and 849 (compared to 189 expected) for the network with primary interactors (*p* < 5e-13, exact Poisson test). The number of interactions per protein (node degree) in the network followed a lognormal right-skewed distribution.

Using this distribution, we detected 10 “protein hubs,” defined as those appearing in the 5% right tail of the node-degree distribution (Supplementary Figures S1A,B) with a node degree higher than 30. Of these 10 hubs, four were *seed* proteins of the TG network: SMAD3, PRNP, HRAS, and PTEN, while the others were primary interactors (Table 4). With the exception of PRNP, which formed a sub-module in the network, the other nine hub proteins shared common interactors (Supplementary Figure S1C). Three hubs of the network are DYRK1A phosphorylation substrates: HRAS, GRB2, and TRP53. Interestingly, the DYRK1A interactors, which include several DYRK1A phosphorylation substrates, had an average node degree of 7.7, significantly higher than the 4.6 average node degree of the network (*p* < 0.01, permutation test). A total of 46 synaptic and 34 neurogenesis-related proteins map on the network, of which PTEN is a hub, and SYNJ1 and MAP1B are known DYRK1A phosphorylation targets. SELENOF, TXN1, and HB-BT belong to the antioxidant activity category.

**TABLE 4:** List of proteins with higher number of interactions.

**Symbol**	**Interactions (*n*)**	***p* values**	**Rescued**	**Seed**	**Correlating**	**DYRK1A target**	**Description**
Rps27a	43	0.010					Ribosomal protein S27A
Uba52	40	0.012					Ubiquitin A-52 residue ribosomal protein fusion product 1
Trp53	38	0.013				Yes	Transformation-related protein 53
Ubc	38	0.013					Ubiquitin C
Rac1	34	0.017					RAS-related C3 botulinum substrate 1
Smad3	28	0.025	Yes	Yes			SMAD family member 3
Prnp	25	0.031	Yes	Yes			Prion protein
Hras	24	0.034	Yes	Yes		Yes	Harvey rat sarcoma virus oncogene
Pten	22	0.040	Yes	Yes			Phosphatase and tensin homolog
Grb2	22	0.040				Yes	Growth factor receptor bound protein 2
Med16	18	0.058		Yes			Mediator complex subunit 16
Rela	17	0.064					v-rel reticuloendotheliosis viral oncogene homolog A (avian)
Pik3r1	17	0.064					Phosphatidylinositol 3-kinase, regulatory subunit, polypeptide 1 (p85 alpha)
Hsp90ab1	16	0.071		Yes	Yes		Heat shock protein 90 alpha (cytosolic), class B member 1
Arhgef7	16	0.071	Yes	Yes			Rho guanine nucleotide exchange factor (GEF7)
Ripk1	15	0.079	Yes	Yes			Receptor (TNFRSF)-interacting serine-threonine kinase 1
Smad4	15	0.079					SMAD family member 4
Pik3ca	15	0.079					Phosphatidylinositol 3-kinase, catalytic, alpha polypeptide
Irs2	14	0.088		Yes			Insulin receptor substrate 2
Hdac1	14	0.088					Histone deacetylase 1
Ubb	13	0.099					Ubiquitin B
Dvl1	13	0.099					Disheveled segment polarity protein 1
Pik3cb	13	0.099					Phosphatidylinositol 3-kinase, catalytic, beta polypeptide
Axin1	13	0.099					Axin 1
Pip5k1c	13	0.099	Yes	Yes			Phosphatidylinositol-4-phosphate 5-kinase, type 1 gamma
Bcl2	13	0.099					B cell leukemia/lymphoma 2
Raf1	12	0.113					v-raf-leukemia viral oncogene 1
Smurf2	12	0.113					SMAD specific E3 ubiquitin protein ligase 2
Casp3	12	0.113					Caspase 3
Mdm2	11	0.128					Transformed mouse 3T3 cell double minute 2
Ralbp1	11	0.128	Yes	Yes			ralA binding protein 1
Pdgfrb	11	0.128					Platelet-derived growth factor receptor, beta polypeptide
Pik3cd	11	0.128					Phosphatidylinositol 3-kinase catalytic delta polypeptide
Psmd4	11	0.128					Proteasome (prosome, macropain) 26S subunit, non-ATPase, 4
Itsn1	11	0.128					Intersectin 1 (SH3 domain protein 1A)
Cav1	10	0.147		Yes			Caveolin 1, caveolae protein
Pik3cg	10	0.147					Phosphoinositide-3-kinase, catalytic, gamma polypeptide
Gja1	10	0.147	Yes	Yes			Gap junction protein, alpha 1
Hsph1	10	0.147	Yes	Yes	Yes		Heat shock 105 kDa/110 kDa protein 1
Nck2	10	0.147	Yes	Yes			Non-catalytic region of tyrosine kinase adaptor protein 2
Prkcd	10	0.147		Yes			Protein kinase C, delta
Synj1	10	0.147	Yes	Yes	Yes	Yes	Synaptojanin 1

*The first 10 were considered “hubs,” with the probability to encounter a protein with a higher number of interaction being < 0.05.*

### Green Tea Extracts Rescue NOR in Mice Overexpressing *Dyrk1A*

**FIGURE 3:**
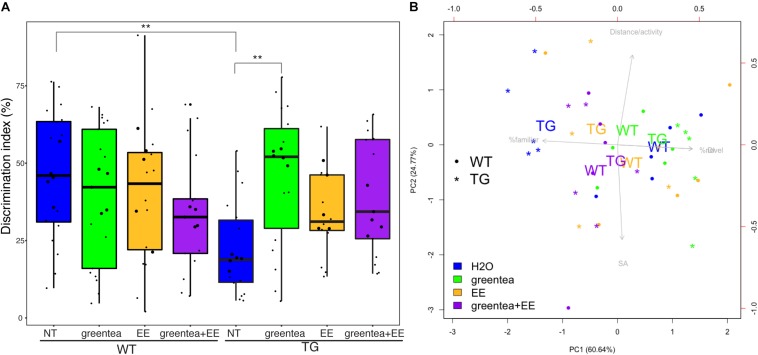
Treatment with green tea extract rescues TG cognitive deficit in the novel object recognition task. **(A)** Boxplots showing the distribution of the discrimination indexes for each group measured during the novel object recognition (NOR) task. The actual values are represented as black dots. Samples selected for proteomic analyses are printed as larger dots. ^∗∗^ *p*-values < 0.01, one-way ANOVA. **(B)** Biplot showing the first two principal components (PC1 and PC2) from NOR data. The percentage of variance explained for each principal component is reported in brackets. Arrows represent the contributions of specific variables to each principal component being the length and orientation of the arrows proportional to their contribution to PC1 and PC2. For the sake of clarity, only the centroids for each group of biological replicates are reported (the averages of PC1 and PC2 values of mice belonging to the same group), represented as WT (wild type mice) and TG (transgenic mice) together with the five samples selected for the (phospho-)proteomics (as dots or stars). “% Familiar” is the percentage of time exploring the familiar object, and “% Novel” is the percentage of time exploring the novel object. Blue: untreated mice; green: treatment with green tea extract; yellow: EE (environmental enrichment); purple: combined treatment.

Once we had defined the phosphoproteome alterations driven by *Dyrk1a* overexpression in TG hippocampus, we studied the effects of three pro-cognitive therapies in TG and WT mice on object recognition memory in order to ensure that possible (phospho-)proteomic changes reflect a pro-cognitive effects in a hippocampal-dependent memory task: (i) green tea extract, which we previously reported to rescue cognitive impairments in the NOR task in transgenic *Dyrk1A* mice (TG) (Martinez de Lagran et al., 2012); (ii) EE, which we previously showed to enhance cognition and reduce *Dyrk1a* expression (Pons-Espinal et al., 2013); and finally (iii) the combined treatment with green tea and EE (Figure 3A).

We here showed that the impaired novelty discrimination in TG mice (*p* = 0.001, one-way ANOVA) was rescued by treatment with green tea extract (*p* = 0.002, one-way ANOVA) as previously described. Treatment with EE and the combined treatment (*greentea* + *EE*) showed only a trend to recovery (*p* = 0.368 and *p* = 0.055, respectively, one-way ANOVA). In WT mice, none of the treatments produced any statistically significant effect. However, when performing the same statistical test using only the mice used for the (phospho-)proteomic experiment (that did not include outliers), we did obtain significant results for each of three treatments in TG (*TG.greentea-TG-NT*, *p* < 0.001; *TG.EE-TG-NT*, *p* < 0.005; *TG.greentea* + *EE-TG.NT*, *p* < 0.05).

A PCA using the variables from the NOR task revealed that learning-related variables (DI and the percentage of time exploring the familiar or novel object) contributed to the first principal component (PC1) and explained a large proportion of the variance (60.6%). On the other hand, the second principal component (PC2) explained 24.8% of the variance and was mainly loaded by spontaneous locomotion and shift-search strategy. Untreated TG mice clustered on the left side of the biplot showing the lowest values of PC1 (learning-related variables), while WT mice clustered on the right. Interestingly, all the treatments promoted transgenic mice to cluster around a higher value of PC1, suggesting a partial rescue of the learning deficit. The transgenic mice treated with green tea extract were the closest to the untreated WT mice with very similar PC1 values, indicating rescued novelty recognition (Figure 3B).

### Antioxidant Activity and Synaptic- and Neurogenesis-Related Pathways Are Rescued by Pro-cognitive Treatments in the Transgenic DYRK1A Phosphoproteome

**FIGURE 4:**
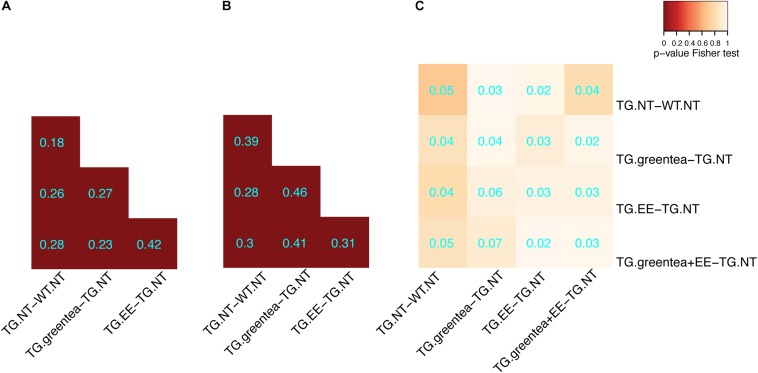
Overlap of the differentially abundant and differentially phosphorylated proteins in transgenic mice after different treatments. **(A)** Heatmap showing the overlap between differentially abundant proteins across the different treatments in transgenic mice. The color code goes from very low *p*-values (red) to high *p*-values (yellow) of the exact Fisher test. TG, TG mice. WT, wild type mice. NT, not treated. EE, environmental enrichment. The Szymkiewicz–Simpson overlap coefficient is printed in cyan. **(B)** Overlap between differentially abundant phosphopeptides across the different treatments. **(C)** Overlap between differentially abundant proteins and proteins with differentially abundant phosphopeptides.

Once we confirmed the positive cognitive effects of our treatments, we analyzed the changes on the hippocampal (phospho-)proteomic profile. We first focused on understanding to what extent the alterations observed when comparing TG to WT mice were rescued. The proteins changing their abundances and/or phosphorylation in the TG hippocampus exhibit a significant overlap with the changes detected upon treatment (contrasts ranging from 18% to 46%; *p*-value Fisher’s test < 0.05) (Figures 4A,B). This shows that green tea extract, EE, and their combination act on targets altered in TG hippocampus, namely, antioxidant, neuronal plasticity, and synaptic proteins. Similar to what was observed for *Dyrk1A* overexpression, upon the different treatments, the changes in protein abundances showed minimal overlap with proteins changing their phosphorylation (Figure 4C), indicating differential effects on the proteome and phosphoproteome.

**FIGURE 5:**
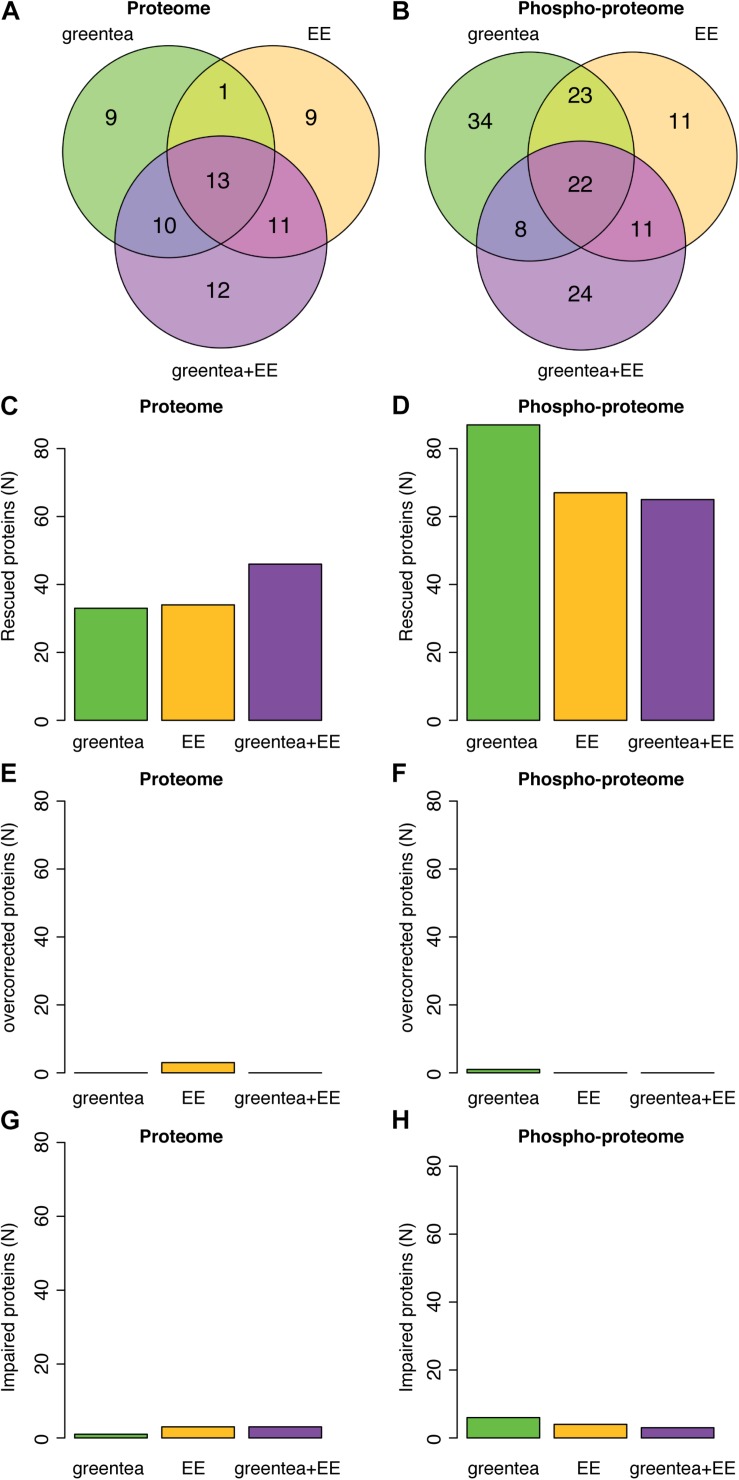
(Phospho-)proteomic alterations in transgenic mice are partially restored by the treatments. **(A)** Venn diagrams showing the overlap of proteins that exhibit restored (rescued) abundances after the treatments. **(B)** Same as in **A** but for the phosphopeptides. Barplot showing the number of rescued proteins **(C)** or phosphopeptides **(D)**, overcorrected proteins **(E)** or phosphopeptides **(F)**, and impaired proteins **(G)** or phosphopeptides **(H)**, after each of the tested treatments.

To identify potential mechanisms explaining the pro-cognitive effects of the different treatments, we analyzed whether proteins and phosphoproteins deregulated in TG were restored to WT levels. We defined as “rescued” those proteins whose abundances and/or phosphorylation levels were partially or completely restored to WT untreated values by at least one of the treatments (Tables 2, 3 and Supplementary Table S21; Figure 5 and Supplementary Figure S2; for more details, see section “Materials and Methods”). At the proteome level, we detected 53 proteins rescued by the single treatments with green tea extract and/or EE (33 and 34, respectively), with 14 proteins commonly targeted by both treatments (Figure 5A). The combined treatment (green tea + EE) rescued half of the proteins modified by either EE or green tea and 12 additional targets not modified by the single treatments. This corresponds to 65 rescued proteins out of the 98 proteins with deregulated abundances.

Similarly, when analyzing the rescued phosphopeptides (Figure 5B), 109 phosphopeptides (belonging to 103 proteins) were rescued by the single treatments with green tea extract and/or EE, of which 42 (39 proteins) were specific to green tea and 22 (22 proteins) were specific to EE, while the rest were rescued by both. The combined treatment rescued 40% of these phosphopeptides and an additional 24 phosphopeptides (15 proteins). Remarkably, this corresponds to 118 out of 190 deregulated proteins whose phosphorylation is rescued. The green tea extract was the treatment rescuing more phosphosites, while *greentea* + *EE* was more effective at the level of protein abundances (Figures 5C,D). Interestingly only a small fraction of proteins (around 9%; < 10 proteins, overall) was “overcorrected” or showed an even bigger difference after treatment when compared to “physiological” WT levels (Figures 5E–H).

A GO-term analysis of the proteins rescued by the treatments showed significant enrichments in antioxidant activity for proteins whose abundance was rescued and synaptic- and neurogenesis-related pathways for proteins whose phosphorylation levels were rescued (Supplementary Figure S3 and Supplementary Table S22). We then mapped these rescued proteins in our network. Around 70% of the *seed* (phospho-)proteins in the network were rescued by at least one of the treatments (blue nodes; Figure 2). Only 9% were not rescued, or further impaired (green nodes). Interestingly, while the rescued (phospho-)proteins had a node degree similar to the average *seed* proteins (4.6), those not rescued by any of the treatments had a significant lower node degree (2.8; *p*-value < 0.001, permutation test). Of note, the four *seed* proteins that are hubs of the network—SMAD3, PRNP, HRAS, and PTEN—were rescued by at least one of the treatments (Table 4).

### DYRK1A Interactors Are Enriched in Differentially Phosphorylated Proteins and Their Interactors

**FIGURE 6:**
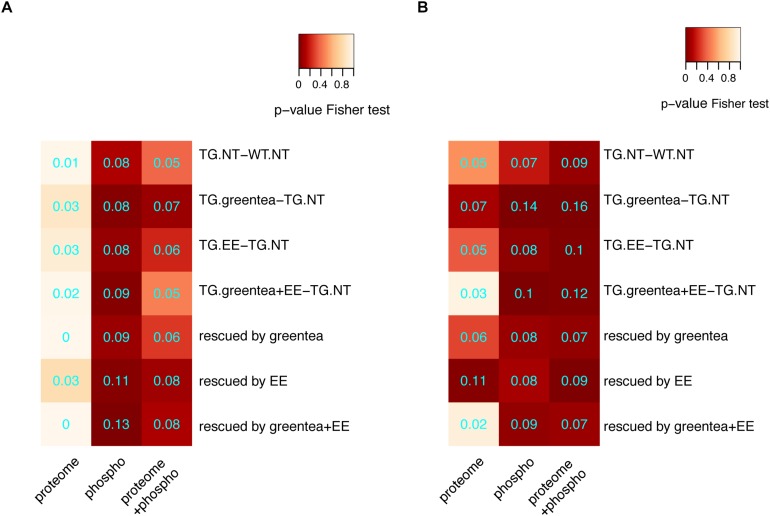
Enrichment for DYRK1A interactors. **(A)** Heatmap showing the *p*-values (Fisher exact test) corresponding to the enrichment of DYRK1A interactors when considering the main contrasts and rescued proteins for proteomic data, phosphoproteomic data, or both lists merged. **(B)** The same as in **A** but with lists extended to the direct interactors. Overlap coefficients are printed in cyan.

We tested the number of DYRK1A interactors (a subset of which could be DYRK1A phosphorylation substrates) in our (phopho-)proteome. Interestingly, we detected higher overlaps in the phospho-proteome where we did detect significant enrichments in the phosphorylation sites of protein changing upon green tea extract treatment (whether combined or not with EE), but not upon EE only. We then specifically analyzed the overlap of DYRK1A interactors with the rescued proteins and we detected statistically significant enrichments for DYRK1A interactors in the phosphopeptides rescued by EE and the combined treatment, but not green tea alone (Figure 6A). Noteworthy, when adding the list of the direct interactors from *STRINGdb* to each of our lists, the overlap between significantly changing (phospho-)proteins and DYRK1A interactors was generally more significant (Figure 6B), since many direct interactors in our network were also DYRK1A interactors.

subsectionPhosphorylation Levels Correlate With Cognitive Performance More Than Protein Abundances

First, we calculated the correlations of all proteins and phosphopeptides obtained from the proteomic experiments with a cognitive score, using an unsupervised analysis. We used the first principal component of our PCA [PC1 values in Figure 3B for the mice selected for the (phospho-)proteome] as a composite measure (cognitive score) of the learning-related variables of the NOR task.

**FIGURE 7:**
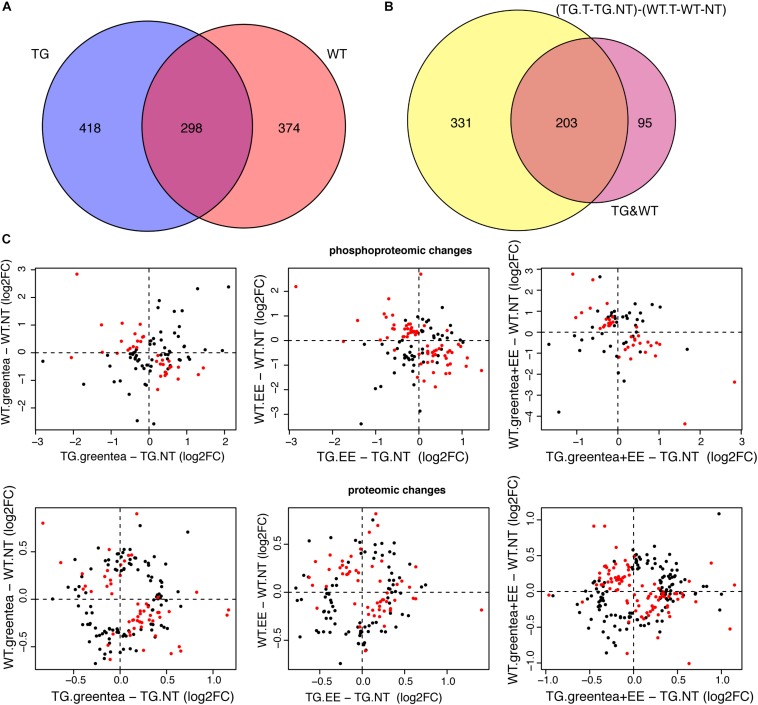
Proteins correlating with cognition. Heatmap showing the *p*-values (Fisher exact test) of the overlaps between proteins correlating with the cognitive variables (PC1 values) and the (phosho-)proteins altered in the genotype contrast, in each of the tested treatments, and in the rescued proteins. The overlap coefficient is printed in cyan. Color code as in Figure 2.

By setting a threshold of |*rho*| > 0.4, we obtained 94 proteins and 205 phosphoproteins that correlated with the cognitive score. While the 94 proteins correlated with PC1 were not significantly overlapping with those changing upon *Dyrk1a* overexpression, the 205 phosphopeptides correlated with the cognitive score were enriched in proteins showing phosphorylation changes upon *Dyrk1a* overexpression. The same profile was detected when analyzing the overlap with the treatments. Also, in those cases, the overlap of the phosphoproteins containing the 205 correlating phosphopeptides was much higher than the overlap with the 94 correlating protein abundances (Figure 7).

Finally, we also analyzed the subset of rescued proteins, confirming that correlating phosphopeptides were significantly enriched in rescued phosphoproteins with four phosphopeptides negatively correlating with the learning-related variables (PC1), and 32 phosphopeptides positively correlating, whereas the abundances of only two rescued proteins negatively correlated with cognition (Figure 7 and Supplementary Figure S2).

In the network, about 90% of the correlating (phospho)-proteins were connected with the hubs in our network, with shortest paths ranging from one to six edges (70% of these paths being shorter than 2).

### DYRK1A-Independent Effects Are Not Enriched in Specific Functional Categories

When computing our rescued proteins, we only focused on the proteins whose abundances or phosphorylation was compromised upon *Dyrk1A* overexpression. However, the treatments had a much wider effect. To capture the overall effect of the treatment, we created a list of all the proteins affected by at least one of the treatments (green tea extract, EE, and their combination), including those altered upon *Dyrk1A* overexpression (183 of the 262), but also 533 that were not altered upon *Dyrk1A* overexpression and so did not belong to the rescued proteins. This indicated that the treatments were not only restoring the portion of the proteome altered in TG mice, but had a much wider effect, affecting a total of 716 proteins. The enrichment analysis detected categories related with “synapse,” similar to what we found for the subset of the rescued proteins (see above). Interestingly, these enrichments were lost when removing the rescued proteins, indicating that the non-rescued proteins were not enriched in any specific function for themselves (Supplementary Figure S4 and Supplementary Table S23).

### The Effects of Green Tea Extract, EE, and Their Combination Are Genotype Specific

Since we observed a different effect in the NOR for WT and TG mice, we assessed if there was a genotype-dependent (phospho-)proteome response to the different treatments. In WT, over 70% of the nodes in the network were modified by the treatment(s) toward TG levels (Supplementary Figure S5, dark green nodes), and thus could be interpreted as deleterious. Noteworthy, the fraction of these “impaired” proteins, with abundance levels being shifted in WT mice toward TG levels by the treatments (dark green nodes in Supplementary Figure S5), was enriched in synaptic pathways. As in TG, also in WT, the treatments were affecting far more proteins than those altered by DYRK1A overdosage, with a total of 672 proteins changing their abundance or phosphorylation. GO-term analysis of these proteins was enriched not only in synaptic categories, as in TG mice, but also in categories related to neurogenesis, cytoskeleton, neuron projections, and GTPase activity, suggesting a broader effect of the treatment(s) in WT mice (Supplementary Figure S4 and Supplementary Table S23). When repeating the enrichment analysis removing the “impaired” proteins from the list of 672 proteins, we found no significant enrichment in any category, meaning that the “impaired” proteins were the most functionally annotated in the treated WT (Supplementary Figure S5 and Supplementary Table S23).

**FIGURE 8:**
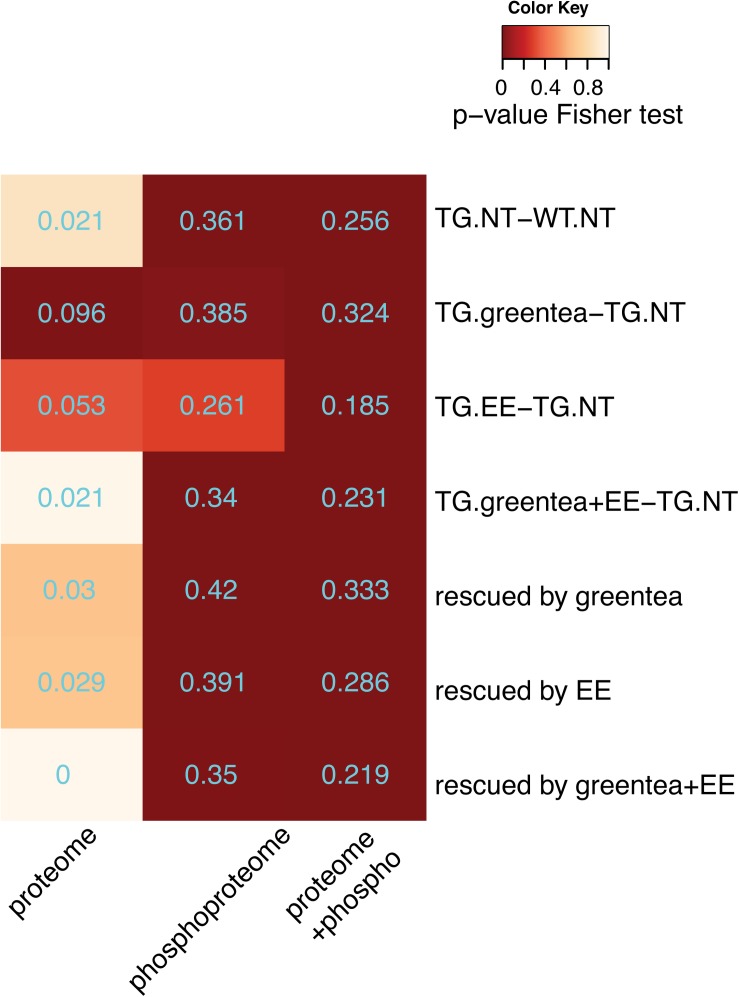
The effects of green tea extract, EE, and the combined treatment are genotype specific. **(A)** Venn diagram showing the overlap between proteins changing their abundance or their phosphopeptide levels upon any of the treatments in TG and wild-type mice. **(B)** Venn diagram showing the overlap between the (phospho-)proteins having the same behavior upon one of the treatments in TG mice and WT mice, and (phospho-)proteins exhibiting a genotype-specific response to treatments. **(C)** Plot comparing phosphopeptide (top panel) and protein (bottom panel) fold changes upon each of the three treatments in TG mice (*x*-axis), and wild-type mice (*y*-axis). Protein or phosphopeptides with a significant interaction in the contrast *(TG.T-TG.NT) - (WT.T-WT.NT)*, where T stands for one of the treatments, and NT for “not treated,” are indicated as red dots.

Of the 672 proteins responding to treatment(s) in WT, 298 were common to the ones responding to treatment(s) in TG (Figure 8A, > 44% overlap, *p* = 3e-49, Fisher exact test), and since they included, 30–34% of impaired/rescued proteins (respectively) were enriched in the same GO-term categories found when analyzing the overall proteins responding to treatment(s) in WT or TG (Supplementary Figure S5 and Supplementary Table S23). We then wanted to verify if these commonly affected proteins were affected similarly in WT and TG mice in terms of both direction (up or down) and fold change of deregulation. Therefore, we compared the changes in abundances of proteins and phosphopeptides in TG mice upon treatment (*TG.T-TG.NT*) with those observed in WT mice (WT.T-WT.NT) [interaction contrast, “(*TG.T-TG.NT)-(WT.T-WT.NT)*”]. Overall, we found that 534 proteins were responding significantly differently to the treatment(s) in TG compared to WT mice. These (phospho-)proteins were enriched in categories including “synapse-related” and other neuronal components, as well as in categories associated with learning and memory (Supplementary Figure S4 and Supplementary Table S23). Of these 534 proteins, 203 were responding to treatment(s) both in WT and TG mice [68% of the 298 proteins commonly affected by the treatment(s) in WT and TG; Figure 8B]. Interestingly, when plotting the fold changes for each treatment in WT versus TG, we found out that most proteins and phosphopeptides differentially regulated by the treatment(s) in TG showed opposite changes in WT (red dots in the top-left and bottom-right portion of the plots, Figure 8C). These proteins included the proteins of the network that were at the same time impaired in WT and rescued in TG mice by the treatments (67% of the rescued proteins in TG mice were impaired in WT mice).

## Discussion

We found that *Dyrk1A* overexpression had a significant impact on protein abundances but produced more pronounced changes in the phosphoproteome in TG hippocampus. These (phospho-)proteomic perturbations mainly involved pathways related to neuronal plasticity and synaptic function and were restored by pro-cognitive therapies such as green tea and/or EE through partially overlapping mechanisms.

We detected 26% of the proteins and 14% of the detected phosphopeptides to be differentially regulated in TgDyrk1A, in agreement with a recent study on a different strain (mBACTgDyrk1A) of mice overexpressing *Dyrk1A* (finding 30% and 23%, respectively; Nguyen et al., 2018). We found almost no overlap between proteins significantly changing abundance and proteins changing phosphorylation (Figure 4C), thus indicating that *Dyrk1A* overexpression has independent effects on the proteome and phosphoproteome. Gene ontology analysis showed that proteins changing in abundance in TG mice were related with antioxidant activity, and proteins with altered phosphorylation sites were enriched in synaptic, neuronal, and neurogenesis-related categories. This observation is relevant given that these categories reflect most of the processes altered in the brain of transgenic mice and include synaptic and neuronal categories (Fernandez et al., 2009).

The effects on protein abundance could be indirect, by activating transcription factors or proteins involved in epigenetic mechanisms (De Toma et al., 2016). Interestingly, we found 10 transcription factors that could explain the protein abundance changes observed in TG mice (Table 1), including FOXO1 that has been described as a DYRK1A substrate (Woods et al., 2001; Yang et al., 2001). In addition, DYRK1A promotes both histone acetylation and deacetylation by phosphorylating SIRT1 (Guo et al., 2010), and CREB transcription factor, respectively (Yang et al., 2001). It also interferes with chromatin remodeling by binding nBAF and reducing the levels of the NRSF/REST neuron-restrictive silencing factor (Lepagnol-Bestel et al., 2009). Moreover, it synergizes histone acetylation by phosphorylating H3 (Himpel et al., 2000) and inhibits HP1 binding (Lepagnol-Bestel et al., 2009).

We used the (phospho-)proteins altered upon *Dyrk1A* overexpression to define a PPI network of proteins altered in TG hippocampus. These *seed* proteins were more connected than expected by chance, indicating their participation in common molecular processes. We detected only few DYRK1A interactors in the *seed* proteins that were deregulated at the level of abundance—such as HRAS—while more than a dozen among the phosphoproteins—including proteins involved in synaptic processes such as MAP1B, ABLIM1, and SYNJ1.

When we included primary *bona fide* interactors in our list of differentially abundant/phosphorylated and rescued (phospho-)proteins, several of these were also DYRK1A interactors. We also detected four DYRK1A interactors among the hubs of the network: two were *seed* proteins, HRAS and SMAD3, and two were primary interactors, TP53 and GRB2. As a matter of fact, DYRK1A phosphorylates p53 participating in its regulation (Park et al., 2010) and interacts with SMAD proteins probably influencing the TGF-β signaling (Brown et al., 2008); moreover, it forms complexes with the GTPase HRAS, enhancing the MAPK cascade (Kelly and Rahmani, 2005), and with GRB2, an adaptor protein of the RAS pathway, which is involved in the ERK signaling (Abekhoukh et al., 2013).

### *Dyrk1A* Overexpression Deregulates Proline-Directed Kinases

When analyzing the phosphopeptides upregulated in our TG mice, we found that almost 79% were phosphorylated on serine residues, 18% on threonine, and only 3% on tyrosine, in agreement with a previous study (Nguyen et al., 2018). We identified the consensus motif PXSP as the most represented tetrapeptide, indicating an alteration of the activity of proline-directed kinases in TG mice. PXSP is a known consensus motif for the ERK1/2/MAPK pathway, which has been linked with learning and memory (46). The analysis of the phosphoproteome led to the identification of two phosphorylated peptides that exactly matched a known phosphorylation motif of DYRK1A (*RPX(S/T)P*) (47). Similarly, a previous study also found only one peptide exactly matching this consensus motif (Nguyen et al., 2018). In our dataset, one peptide belonged to SGIP1 (SH3-containing GRB2-like protein 3-interacting protein 1), a protein that mediates clathrin endocytosis and interacts with some DYRK1A interactors essential to the formation of functional clathrin-coated pits in neurons, such as dynamin 1, amphiphysin 1, endophilin, or synaptojanin 1. Overexpression of *Dyrk1A* impairs clathrin-mediated endocytosis in fibroblasts and neurons by reducing the co-localization between dynamin and clathrin at the plasma membrane leading to delays in vesicle internalization and thus synaptic malfunctions (Kim et al., 2010). Our findings reveal SGIP1 as a putative new DYRK1A target that could explain DYRK1A-mediated endocytosis impairment.

The second phosphopeptide with the *RPX(S/T)P* motif belonged to SHANK3, a postsynaptic density protein with a role in synaptic plasticity that acts as a scaffold protein to bind NLGN-NRXN and NMDAR at the postsynaptic density and has been involved in intellectual disabilities and autism. Given that DYRK1A also localizes in postsynaptic zones, it is plausible that phosphorylation of SHANK3 could occur through DYRK1A (Arque et al., 2013).

### Green Tea Extract, EE, and Their Combination Partially Rescue *Dyrk1A* Overexpression and Proteomic and Phosphoproteomic Alterations

In our experiments, 1 month of treatment with green tea extract rescued the memory impairment in the NOR test in TG mice (Martinez de Lagran et al., 2012). This reproduces previous results that published recovery in both the NOR and Morris Water Maze test (De la Torre et al., 2014). Both EE and the combined treatment also showed a trend to improve NOR test performance in TG mice, but in this case, the effect did not reach statistical significance (Figure 3A). This can be because EE can trigger stress in mice, especially when using male mice due to their more aggressive behavior (Haemisch and Gartner, 1997). This is supported by a previous study that showed that trisomic female mice improved their performance in the Morris Water Maze upon EE while male mice decreased their performance (Martinez-Cue et al., 2002). Even though we minimized this problem by rearing the mice together before sexual maturity, that in the past allowed EE to be effective also in male trisomic mice (Begenisic et al., 2011), this factor cannot be totally excluded. However, when performing the same statistical test using only the mice used for the (phospho-)proteomic experiment, we did obtain significant results for each of three treatments (*TG.greentea-TG-NT*, *p* < 0.001; *TG.EE-TG-NT*, *p* < 0.005; *TG.greentea* + *EE-TG-NT*, *p* < 0.05), suggesting that maybe we lacked the power to detect the lower effect of EE and greentea + EE in the full dataset (since the estimates were lower compared to green tea).

Most proteins changing their abundance or phosphorylation state upon *Dyrk1A* overexpression were significantly modified upon treatments. This observation suggests that green tea, EE, or their combination are acting on a significant subset of proteins/phosphoproteins altered in the network (Figure 4).

Seventy percent of the nodes belonging to the network were rescued (Figure 2) while only few proteins were overcorrected, or further impaired (green nodes in Figure 2). Rescued proteins can be considered key components of the network since most had higher number of interactions (average node degree of 4.1) compared to the non-rescued proteins (average node degree of 2.8). As such, each of the four *seed* protein hubs of the network was rescued by one or more of the treatments.

Noteworthy, GO-term enrichment analysis revealed that rescued proteins pertain to the same pathways and functional processes of the proteins affected by *Dyrk1A* overexpression (Supplementary Figure S3). Rescued proteins were enriched in antioxidant-related GO-term categories, while proteins with rescued phosphorylation levels were enriched in synaptic and neurogenesis proteins. The lack of balance in the metabolism of free radicals generated during processes related to oxidative stress may have a direct role in producing the neuropathology of DS including its comorbidity with Alzheimer’s disease (Hajjar et al., 2018). Green tea treatment was found to affect proteins involved in oxidative stress in a previous study in DS (Valenti et al., 2013), which may be linked to its pro-cognitive properties (De la Torre and Dierssen, 2012). We then analyzed the possible treatment-specific and overlapping effects of green tea extract and EE. All the treatments (green tea extract, EE, or their combination) affected a similar subset of proteins (Figure 4), with 54% of the rescued proteins (medium blue in Figure 2) shared by at least two treatments and 22% shared by the three treatments (dark blue in Figure 2), indicating overlapping mechanisms of action. However, the green tea extract had a much wider effect on the phosphoproteome than EE or the combined treatment. The fact that our treatments’ combinations show both overlapping and unique effects is very interesting since green tea extract dosage could be scaled down when used in combination with another treatment; however, further studies are needed to optimize new effective therapeutic strategies.

Of course, it would be very important to control also for age. As a matter of fact, studies with different formulations containing EGCG show different effects depending on the age. For example, the latency to reach the escape platform was rescued by green tea extracts containing EGCG in young (3 months) TgDyrk1A mice but not in older (5–6 months) mice (De la Torre et al., 2014; Catuara-Solarz et al., 2015, 2016).

Common rescued proteins and phosphoproteins by all treatments were involved in intellectual disabilities and cognitive decline (Supplementary Table S21). An interesting example is Synaptojanin 1, encoded by chromosome 21, which was phosphorylated exclusively in TG mice and interacted with 10 proteins in the network. Synaptojanin 1 was found upregulated in synaptosome preparations of the Ts65Dn partial trisomic mouse model of DS and its genetic ablation successfully attenuates neurodegeneration in an Alzheimer’s disease mouse model, accelerating beta-amyloid clearance (Fernandez et al., 2009). In our experiments, Synaptojanin 1 phosphorylation was rescued by the green tea extract, EE, and the combined treatment, and its phosphorylation level correlated with the performance of mice in results of the cognitive test.

Other rescued proteins were involved in neuronal processes including neurotransmission, synaptic plasticity, neurogenesis, neuronal differentiation, axogenesis, and dendritogenesis (Supplementary Table S21), for example, microtubule-associated protein 1B (MAP1B), a DYRK1A interactor involved in cytoskeletal changes and implicated in neurite extension and synaptic maturation (Scales et al., 2009), and PTEN, a protein phosphatase deregulated in the cortex of DS individuals (Weitzdoerfer et al., 2002) and involved in different signaling cascades including PI3K-AKT, one of the main hubs of the network. PTEN phosphorylation, in a site known to inhibit its phosphatase activity, was only detected in WT mice and upon treatment with the green tea extract in TG mice.

Finally, the prion protein PRNP was also a hub of the network rescued in TG mice that might be involved in neurodevelopment and synaptic plasticity (Steinert, 2015). Other proteins that we found included Synapsin 1 and adducin 2 beta whose phosphorylation was upregulated also in synaptosome preparation of DS mouse models and the β4 subunit of the voltage-dependent calcium channel (Fernandez et al., 2009).

Clinical trials in humans (De la Torre et al., 2016) have shown a better effect when combining green tea and cognitive stimulation in patients, and those results have been validated in the Ts65Dn mouse model (Catuara-Solarz et al., 2016). We thus expected that the combination of green tea extract and EE could potentiate the therapeutic effect of the individual treatments. However, in our experiments, the combination was less effective than the individual treatments. It is worth noting that the green tea extract rescued the phosphorylation levels of more proteins than its combination with EE. While this does not allow discarding an interaction between EE and the treatment with the green tea extract, it does not suggest additive or synergic effects of the treatments since many targets were specific to EE or the treatment with green tea extract. Moreover, these results are difficult to interpret in the context of our behavioral results, since EE treatment can trigger stress in mice, and thus, part of the observed changes may be due to these stress-related responses.

### Phosphorylation Levels Reflect Changes in Cognition

One important limitation of previous proteomic studies was the use of pooled protein extracts from the brains of several animals, which precluded the correlation of the proteomic changes with behavioral outcomes. In our study, behavioral data from the NOR test and the proteomic data were generated on the same mice, so that we could directly correlate the performance in the NOR test, with changes in protein abundances and phosphorylation levels. This analysis revealed that phosphorylation levels were correlating much more with cognition than protein abundance levels. This is relevant since, as discussed above, the phosphoproteome is more affected by *Dyrk1A* overexpression than protein abundances, and the green tea extract—the most effective treatment in improving the DI—rescued more phosphoproteins than EE or the combined treatment. Moreover, correlating proteins were enriched in rescued proteins and were connected with all the hubs in our network.

We speculate that phosphorylation changes that play a main role in cognitive processes such as synaptic and neurogenesis pathways, are impaired in TG mice, and the treatments act by restoring those changes.

### Cognitive Enhancer Effects Are Mediated by DYRK1A-Dependent and Other Independent Mechanisms

The effects of EGCG-containing green tea extracts and EE have been attributed to many different mechanisms such as antioxidant activity, anti-inflammatory, chromatin regulation, and modulation of DYRK1A activity (Pons-Espinal et al., 2013; Valenti et al., 2013; De la Torre et al., 2014; De Toma et al., 2016). Therefore, we assessed whether the observed changes upon treatment with green tea extract and EE were DYRK1A dependent or whether there were also other potential mechanisms involved.

We considered DYRK1A-dependent effects the ones on the proteins in the network, e.g., proteins altered in TG mice by *Dyrk1a* overexpression. In fact, the treatments rescued most of the *seed* proteins of the network, and thus may act through DYRK1A-dependent effects. However, 79% of the proteins modified by the treatments do not belong to the network. This could be interpreted as the treatments potentially acting through other mechanisms not necessarily dependent on DYRK1A. GO-term analysis revealed no significant enrichments for these proteins, suggesting that these treatment-related changes may be unspecific. However, when pooling DYRK1A-independent and rescued proteins, many more categories showed up, including synaptic components and many other categories related to neurogenesis, cytoskeleton, neuron projections, and GTPase activity. This means that even though these proteins, when taken alone, were not specifically enriched in any pathway, many of them belonged to the same pathways enriched for rescued proteins. This suggests that the DYRK1A-independent effects of the treatments “complemented” the effect of the rescued proteins, and therefore might be involved in further “correction” of the phenotype (Supplementary Figure S5).

Our results agree with previous work, in which inhibition of DYRK1A with a pharmacological inhibitor of DYRK1A (leucettine 41) was effective on the NOR score in transgenic mice, affecting cytoskeletal, synaptic, and learning pathways (Nguyen et al., 2018). Interestingly, they found five proteins to be consistently rescued upon treatment in two mouse models (transgenic for *Dyrk1A* and the partial trisomic Ts65Dn) and three cerebral areas (hippocampus, cortex, and cerebellum).

The phosphorylation levels of three of these five proteins were also rescued by our treatments: MAP1A’s phosphorylation was fully rescued by the treatment with green tea extract; MAP2 was fully rescued by the combined treatment, while SYN1, which was found by the same authors to interact directly or indirectly with DYRK1A, was fully rescued by both EE and the combined treatment. This is striking since we obtained results like a pharmacological inhibitor simply by using a nutraceutical such as green tea extract and/or EE. However, it should be pointed out that since we used a green tea extract containing EGCG (45%), we could not disentangle EGCG-specific effects. Most probably, the combined action of EGCG with other compounds in the extracts, such as epigallocatechin (EGC), epicatechin-gallate (ECG), and epicatechin (EC), is required for the pro-cognitive effect as suggested by the fact that EGCG alone (95%) was not able to rescue the cognitive phenotype in the DS mouse model Ts65Dn (Stringer et al., 2015, 2017). The dose of EGCG and the composition of EGCG-containing supplements might be very important as shown also in a previous publication showing differential effects of six commercial supplements containing EGCG on correcting abnormalities in Ts65Dn, comparing two of these formulations to pure EGCG (Abeysekera et al., 2016).

### Green Tea Extract, EE, and the Combined Treatment Have Different Effects in Transgenic and WT Mice

Treatment of WT mice had no significant cognitive effects, although we found a significant overlap in (phospho-)proteins modified by the different treatments in WT and TG mice. However, 68% of these proteins were differentially regulated by the treatment depending on the genotype (Figure 8), with many showing an opposite response (e.g., upregulated upon treatment in TG and downregulated in WT). Interestingly, among these proteins exhibiting a genotype-specific response, we detected enrichments in categories including synapse and other neuronal components and learning and memory. Moreover, in WT mice, 70% of the nodes within the network shifted in abundance or phosphorylation after treatment toward levels like those exhibited by transgenic mice (Supplementary Figure S4). These data point to the importance to fine-tune DYRK1A abundance, without over-correcting its levels. Possibly, in WT mice, this could justify the trend observed in the NOR test for the DI to be slightly impaired upon treatment. Taken together, these results point to a strong interaction between treatment and genotype, as while in TG mice the treatment successfully rescued key network alterations, the treatment in WT mice can be deleterious by certain biological processes related to cognition.

## Conclusion

Our study shows that overexpression of *Dyrk1A* causes broad abundance changes in the proteome and phosphoproteome of the hippocampus of transgenic mice, with most changes affecting proteins related to neuroplasticity and intellectual disabilities. Since more than 70% of the observed changes were rescued by at least one of the tested treatments, our findings suggest that cognitive enhancer treatments can reverse the impairments in conditions of DYRK1A overdosage. Interestingly, the effects of treatments such as EGCG-containing green tea extract and EE are not limited to restoring the protein network affected by DYRK1A overexpression, but extend to many proteins related with neuronal and non-neuronal categories. Moreover, our results establish a specific correlation of cognitive improvement and proteome and phosphoproteome changes. These approaches could be used to develop new combinatorial therapies to boost or prolong current cognitive-enhancement approaches for the treatment of intellectual disabilities. Overall, our data support the idea that DYRK1A activity normalization is critical, and further studies are needed to disentangle the roles of the different components in the green tea extract and to better assess any additive, synergic, and/or antagonistic processes with EE.

## DATA AVAILABILITY STATEMENT

The datasets generated for this study can be found in the proteomic data in PRIDE: PXD010689; a repository with the R markdown file with the code to fully reproduce the analysis is present at the repository: https://bitbucket.org/ilario_de_toma/proteomicsdyrk1a.egcg.ee/src.

## Ethics Statement

The animal study was reviewed and approved by the Comité Ético de Experimentación Animal del PRBB (CEEA-PRBB); MDS 0035P2.

## Author Contributions

ID performed most of the analyses, wrote the manuscript, and prepared all the figures. MO performed the proteomics experiment and made some of the analyses. MD and ES conceived the project and revised the manuscript. PA provided updated protein–protein interactions and revised the manuscript.

## Conflict of Interest

The authors declare that the research was conducted in the absence of any commercial or financial relationships that could be construed as a potential conflict of interest.
