# Supplementary material for: DYRK1A Overexpression Alters Cognition and Neural-Related Proteomic Pathways in the Hippocampus That Are Rescued by Green Tea Extract and/or Environmental Enrichment
Source: Front Mol Neurosci. 2019 Nov 15;12:272. doi: 10.3389/fnmol.2019.00272 (PMC6873902; doi:10.3389/fnmol.2019.00272)
Supplement: Supplementary file 1 [file Data_Sheet_1.zip › Supplementary_Tables_Captions.pdf]

Supplementary table captions:

Supplementary Table 1. *MSstats* results for the WT.NT-TG.NT contrast, showing the Uniprot ID, log2FC, and statistical parameters such as the adjusted p-value for the analysis of the differentially abundant proteins (sheet 1). Sheet 2 and 3 show the proteins exclusively present in one of the conditions of the contrast.

Supplementary Table 2. The same as in Supplementary Table 11 but for the WT.NT-WT.greentea contrast.

Supplementary Table 3. The same as in Supplementary Table 11 but for the WT.NT-WT.EE contrast.

Supplementary Table 4. The same as in Supplementary Table 11 but for the WT.NT-WT_greentea+EE contrast.

Supplementary Table 5. The same as in Supplementary Table 11 but for the TG.NT-TG.greentea contrast.

Supplementary Table 6. The same as in Supplementary Table 11 but for the TG.NT-TG.EE contrast.

Supplementary Table 7. The same as in Supplementary Table 11 but for the TG.NT-TG.greentea+EE contrast.

Supplementary Table 8. The same as in Supplementary Table 11 but for the (WT.NT-WT.greentea) - (TG.NT-TG.greentea) contrast.

Supplementary Table 9. The same as in Supplementary Table 11 but for the (WT.NT-WT.EE) - (TG.NT-TG.EE) contrast.

Supplementary Table 10. The same as in Supplementary Table 11 but for the (WT.NT-WT.greentea+EE) - (TG.NT-TG.greentea+EE) contrast.

Supplementary Table 11. *MSstats* results for the WT.NT-TG.NT contrast, showing the protein ID, and the sequence of the peptide with the probability of phosphorylation of the sites, log2FC, and statistical parameters suh as the adjusted p-value for the analysis of the differentially abundant proteins (sheet 1). Sheet 2 and 3 show the proteins exclusively present in one of the conditions of the contrast.

Supplementary Table 12. The same as in Supplementary Table 11 but for the WT.NT-WT.greentea contrast.

Supplementary Table 13. The same as in Supplementary Table 11 but for the WT.NT-WT.EE contrast.

Supplementary Table 14. The same as in Supplementary Table 11 but for the WT.NT-WT_greentea+EE contrast.

Supplementary Table 15. The same as in Supplementary Table 11 but for the TG.NT-TG.greentea contrast.

Supplementary Table 16. The same as in Supplementary Table 11 but for the TG.NT-TG.EE contrast.

Supplementary Table 17. The same as in Supplementary Table 11 but for the TG.NT-TG.greentea+EE contrast.

Supplementary Table 18. The same as in Supplementary Table 11 but for the (WT.NT-WT.greentea) - (TG.NT-TG.greentea) contrast.

Supplementary Table 19. The same as in Supplementary Table 11 but for the (WT.NT-WT.EE) - (TG.NT-TG.EE) contrast.

Supplementary Table 20. The same as in Supplementary Table 11 but for the (WT.NT-WT.greentea+EE) - (TG.NT-TG.greentea+EE) contrast.

Supplementary Table 21. Summary of the proteins whose abundance and/or phosphorylation is compromised in *TG.NT* mice compared to *WT.NT* mice, which are rescued by at least one of the treatments in TG mice. Both Uniprot IDs, and MGI symbols, and descriptions are provided, and for each proteins the % of recovery as defined in *M&M* is reported (when available). In case of recovery of phosphorylation, the specific phosphosite is reported.

Supplementary Table 22. *ClusterProfiler* report of the Gene Ontology enrichment analysis of the proteins whose abundances and/or phosphorylation is rescued. It reports the GO ID, description, gene ratio compared to the background ratio, p-value, q-value and False Discovery Rate, count of the genes found in each set with their MGI symbols.

Supplementary table 23. The same as in Supplementary Table 22 but with the following set of proteins: 1) Changing in TG upon at least of the treatments; 2) Changing in WT upon at least of the treatments; 3) Changing in both TG and WT upon at least of the treatments; 4) Differently changing upon treatment(s) in TG and WT upon at least of the treatments.
