# Supplementary figures and images for: DYRK1A Overexpression Alters Cognition and Neural-Related Proteomic Pathways in the Hippocampus That Are Rescued by Green Tea Extract and/or Environmental Enrichment
Source: Front Mol Neurosci. 2019 Nov 15;12:272. doi: 10.3389/fnmol.2019.00272 (PMC6873902; doi:10.3389/fnmol.2019.00272)

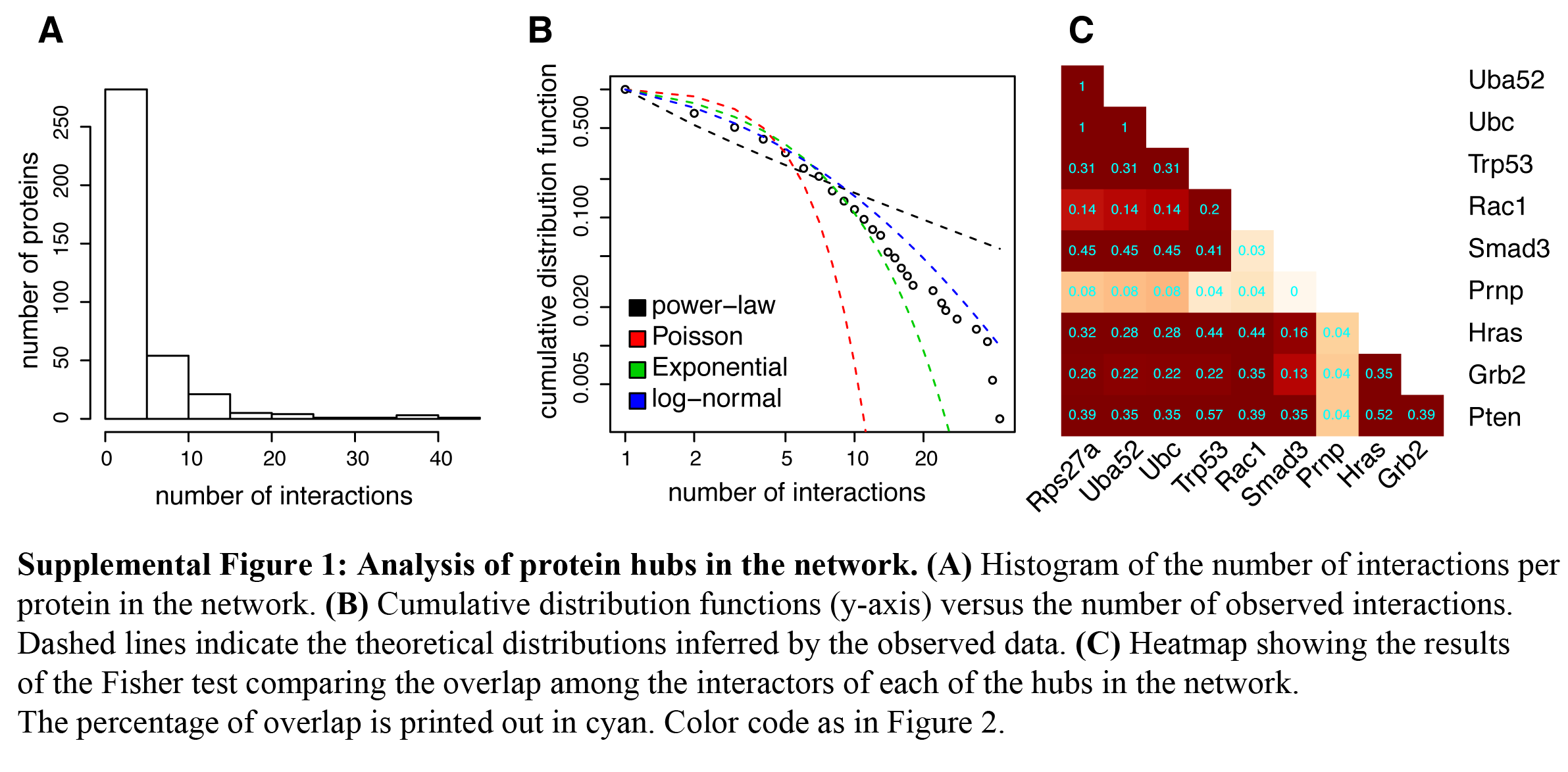

Supplement: Supplementary file 2 [file Image_1.TIF]

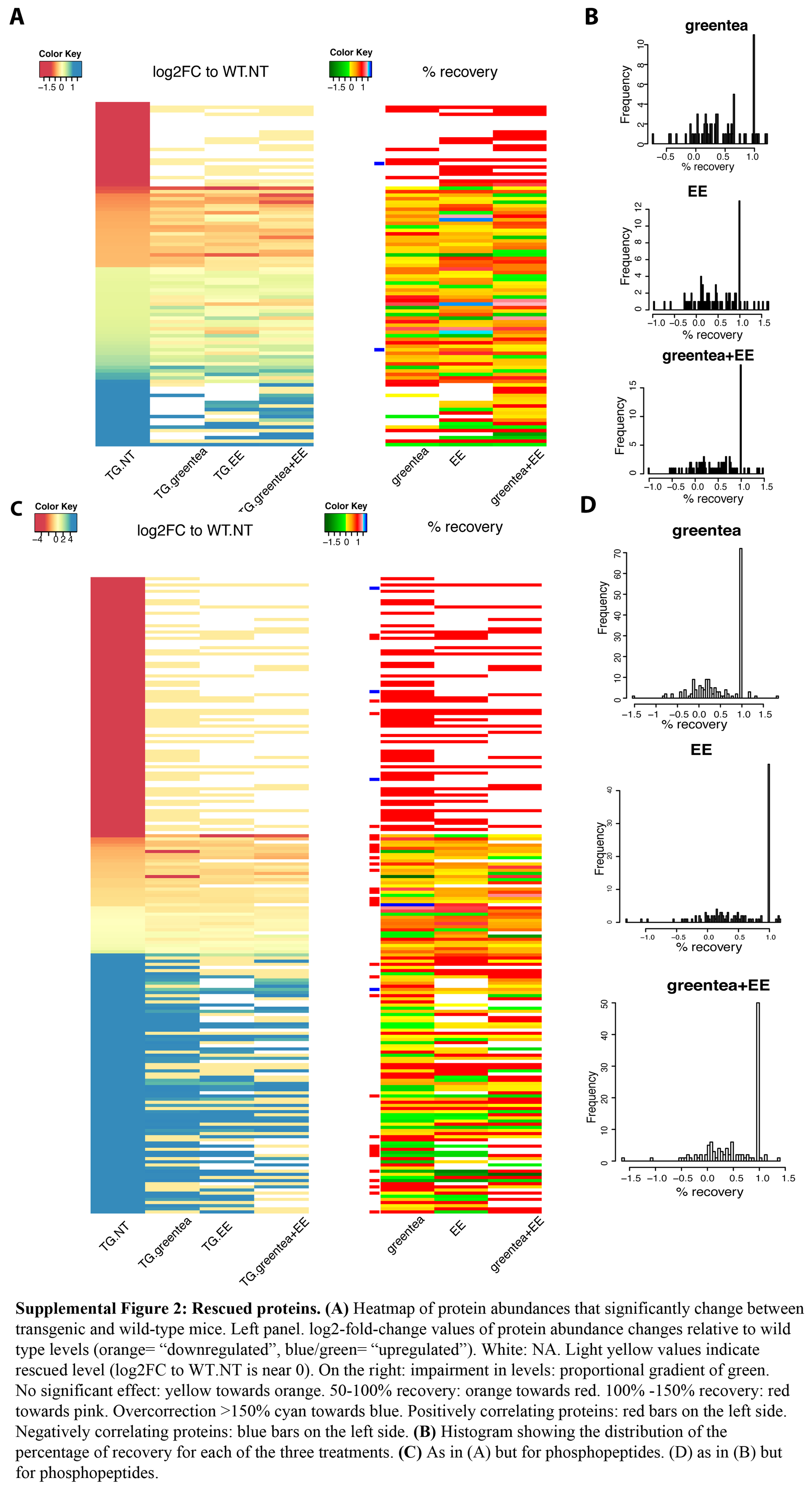

Supplement: Supplementary file 3 [file Image_2.TIF]

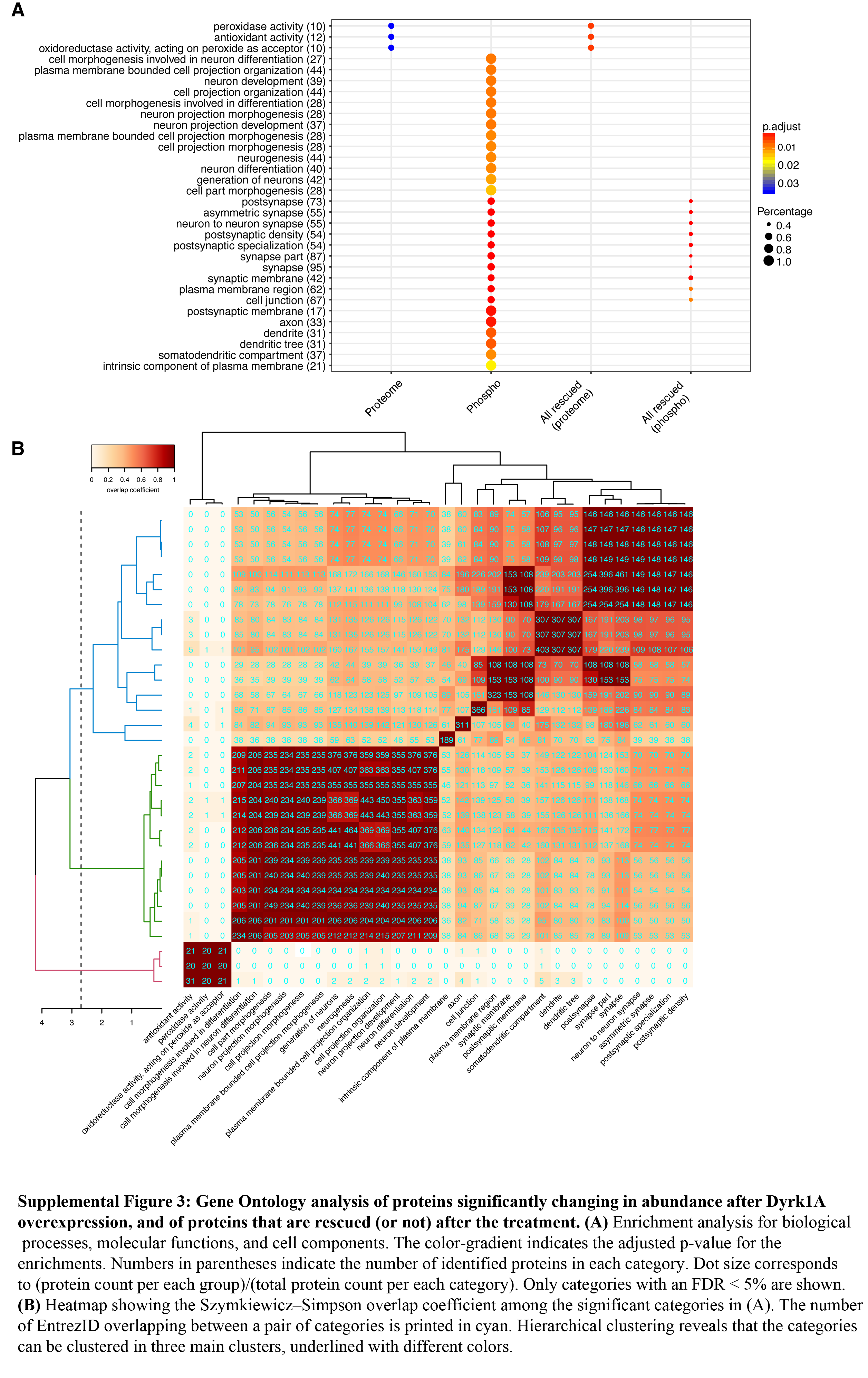

Supplement: Supplementary file 4 [file Image_3.TIF]

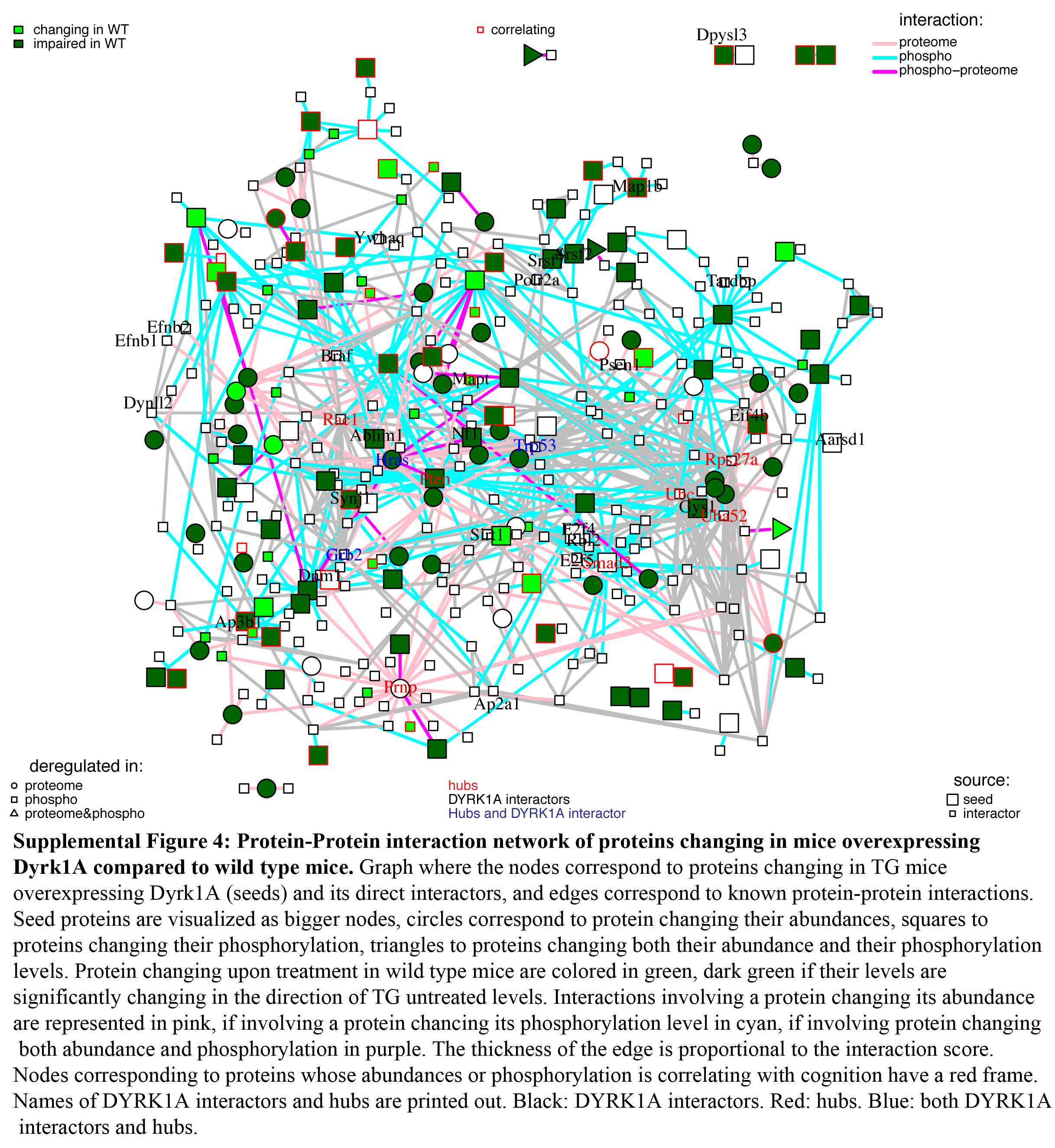

Supplement: Supplementary file 5 [file Image_4.TIF]

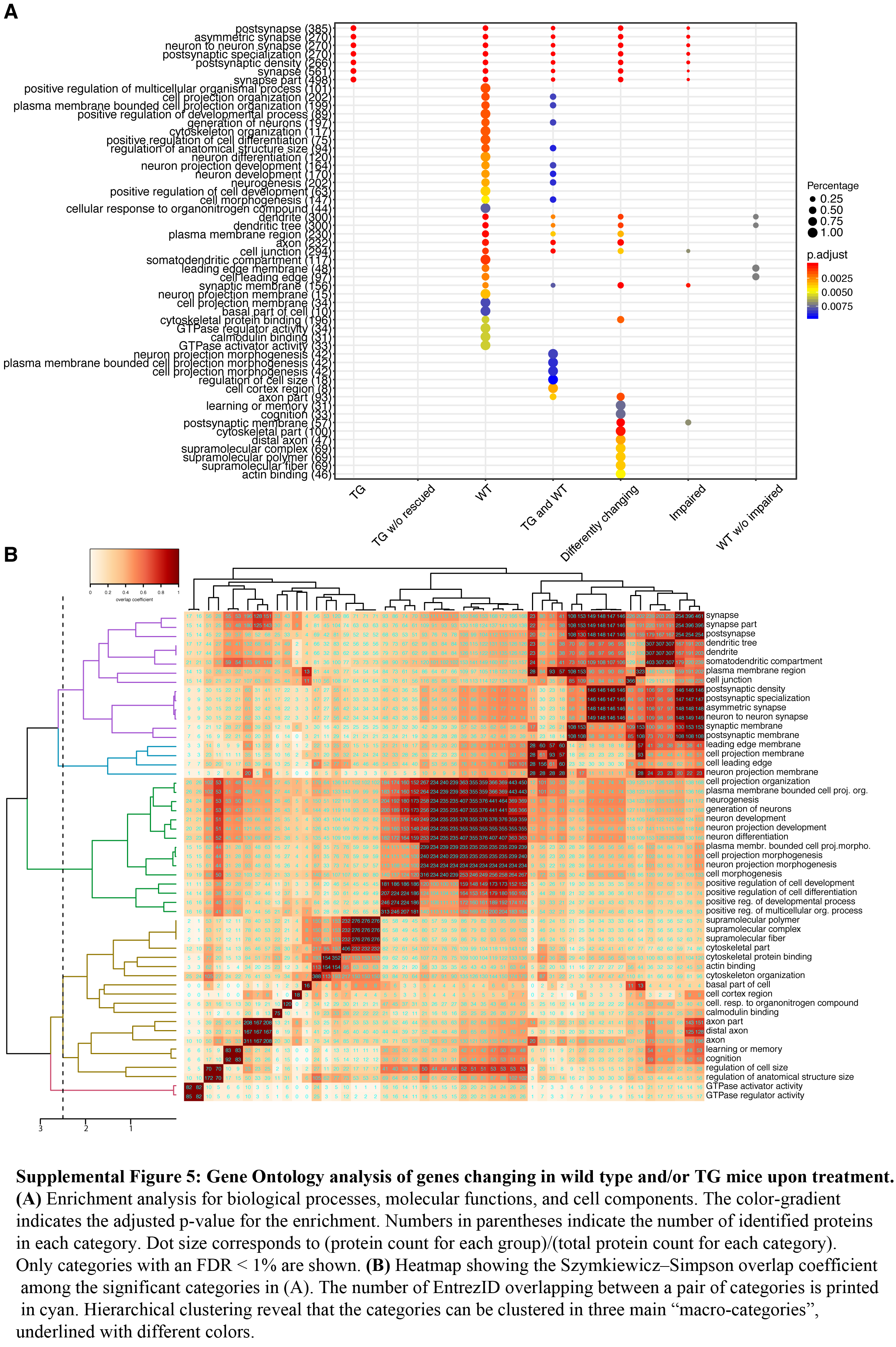

Supplement: Supplementary file 6 [file Image_5.TIF]

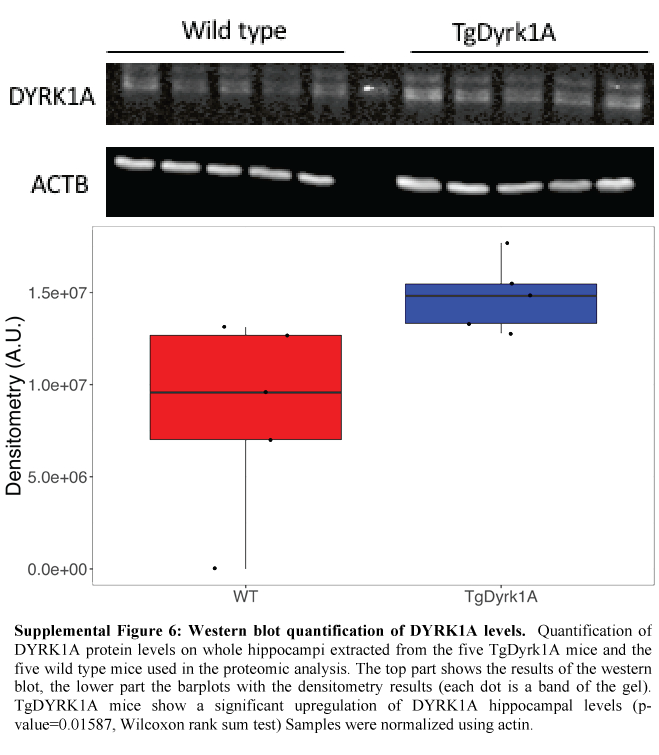

Supplement: Supplementary file 7 [file Image_6.tiff]
